# Role of *Zhiqiao Chuanlian* decoction in the treatment of food accumulation fever: Network pharmacology and animal experiments

**DOI:** 10.1016/j.heliyon.2024.e29813

**Published:** 2024-04-17

**Authors:** Chuxin Zhang, Ruoshi Zhang, Yuli Cheng, Jingpeng Chen, Ruizi Zhu, Lin Gao, Mei Han

**Affiliations:** a Qi-Huang Chinese Medicine School, Beijing University of Chinese Medicine, 100029, Beijing, China; b The Second Clinical Medical College, Beijing University of Chinese Medicine, 100029, Beijing, China; c School of Chinese Materia Medica, Beijing University of Chinese Medicine, 100029, Beijing, China; d School of Traditional Chinese Medicine, Beijing University of Chinese Medicine, 100029, Beijing, China

**Keywords:** Traditional Chinese medicine, Aurantii Fructus, Coptidis Rhizoma, Nitric oxide synthase, Network pharmacology

## Abstract

**Objective:**

Food accumulation fever (FAF), a common clinical disease in children, is generally induced by the excessive intake of high-calorie or high-fat foods. *Zhiqiao Chuanlian* decoction (ZQCLD) is a classical traditional Chinese medicine (TCM) that may have therapeutic effects on FAF.

**Methods:**

Network pharmacological analyses of ZQCLD and FAF were conducted. Animal experiments lasted for 14 days. Rats in the model, positive control, and low-, medium-, and high-dose groups were fed a high-calorie diet. On days 11–14, the positive group was given a domperidone solution. The low-, medium-, and high-dose groups were administered different concentrations of ZQCLD. The body temperature, gastric emptying rate, and intestinal propulsion rate were measured. Relevant indicators were determined by ELISA.

**Results:**

The main target proteins included IL-1β, C–C motif chemokine 2 (CCL2), prostaglandin G/H synthase 2 (PTGS2), transcription factor AP-1 (JUN), haem oxygenase 1 (HMOX1), interferon-gamma (IFN-γ), peroxisome proliferator-activated receptor-gamma (PPAR-γ), and inducible nitric oxide synthase (NOS2/iNOS). Compared with those in the control group, body weight, gastric emptying rate, intestinal propulsion rate, and neuronal nitric oxide synthase (NOS1/nNOS) levels were significantly lower in the model group, whereas body temperature and endotoxin, interleukin-1β (IL-1β), PGE2, and iNOS levels were increased. In each treatment group, body temperature and PGE2 levels returned to normal levels. Compared with those in the model group, the gastric emptying rates in the positive group and the low- and medium-dose groups increased; the intestinal propulsion rates were higher in the medium- and high-dose groups, whereas the endotoxin and IL-1β levels were lower; and the nNOS level was higher in the high-dose group, whereas the iNOS level was lower.

**Conclusions:**

ZQCLD may treat FAF by regulating jejunal IL-1β and nNOS, serum endotoxin, and hypothalamic PGE2 and iNOS levels.

## 1 Introduction

With improvements in living standards in recent years, children's diets have exhibited a trend towards eutrophication. An increasing number of delicious, high-calorie, and high-nutrient foods are available for this purpose. The parents' desire for adequate nutrition for their children may result in a high food consumption by the children [1]. Children may be attracted to the enticing flavours and textures of these foods, leading to excessive intake [[2], [3], [4]]. According to traditional Chinese medicine (TCM) theory, continuous excessive intake of high-calorie or high-fat foods may result in bloating or abdominal pain, hiccups, nausea and vomiting, loss of appetite, constipation, or loose stools, which is called food accumulation (FA). This is similar to the functional dyspepsia (FD) in modern medicine [5]. Owing to an underdeveloped digestive system, children are more susceptible to damage from overeating [6]. This results in FA being more common in children than in adults. Furthermore, a particular phenomenon has been observed in clinical practice in which FA can lead to fever without coinfection. This condition is called FA fever (FAF) and has been documented in ancient TCM books. Because there is no established standard definition or accepted diagnostic criteria, FAF is typically treated as one of two independent disorders: dyspepsia or fever. Consequently, antibiotics, antipyretics, and digestive system stimulants are frequently used to treat the symptoms. However, these separate therapeutic approaches can quickly result in disease recurrence and other gastrointestinal symptoms [7,8]. When FA and FAF are not treated in a timely manner, they may further affect the subsequent growth and development of children [9,10]. Therefore, it is necessary to study FAF, which has been well recorded in ancient texts, but has received little attention in recent years.

Previous animal studies have shown that long-term (>21 days) free intake of high-calorie, high-sugar, or high-fat diets can lead to decreased gastrointestinal motility in animal models [11,12], accompanied by gastrointestinal mucosal barrier damage [13] and local intestinal inflammation [14,15]. Moreover, systemic inflammation may increase [16]. Some short-term experiments (6–21 d) [[17], [18], [19], [20]] revealed that these dietary patterns have an impact on gastrointestinal motility and the gastrointestinal mucosal barrier, accompanied by local intestinal inflammation. However, the impact of short-term but excessive high-calorie dietary intake on young individuals remains unclear. Moreover, the reason for the FA-induced increase in body temperature due to FA in children remains unclear. The present study was conducted to address this issue.

In China, TCM has been used for more than 2000 years. With the advantages of abundant resources, few side effects, stable efficacy, and multiple pathways of action, TCM has shown significant therapeutic potential in many basic and clinical studies [21]. *Zhiqiao Chuanlian* decoction (ZQCLD) is a classical TCM prescription composed of *Aurantii Fructus* and *Coptidis Rhizoma*, which was recorded in ancient texts to relieve FAF caused by consuming excessive amounts high-fat or high-sugar foods. ZQCLD is composed of only two drugs, which avoids potential effects of excessive medication in children and is therefore suitable for the treatment of FAF in children. Previous studies on the effects of *Aurantii Fructus* and *Coptidis Rhizoma* and their underlying mechanisms have shown that *Aurantii Fructus* regulates gastrointestinal motility and alleviates intestinal inflammation [22,23], whereas *Coptidis Rhizoma* has anti-inflammatory and antipyretic properties [24,25]. These studies suggest that ZQCLD may play a therapeutic role by restoring gastrointestinal motility, preventing inflammation, and reducing body temperature. However, this hypothesis remains to be verified.

Network pharmacology is a new branch of pharmacology that uses computer models [26], network analysis methods [27,28] and principles of systems biology to analyse the multi-component, multi-target, and multi-pathway synergistic relationships between drugs, diseases, and targets [29]. The mechanism of TCM prescription involves multiple targets and levels, similar to the integrity, systematisation, and comprehensiveness of network pharmacology. Network pharmacology is a promising method for revealing the potential mechanisms and targets of TCM in disease intervention [30].

Therefore, this study aimed to explore (i) the mechanisms by which FA causes fever and (ii) the therapeutic effects of ZQCLD through network pharmacology analysis and animal experiments to enhance the scientific evidence in support of the TCM theory and provide options for the diagnosis and treatment of FAF in children.

## 2 Materials and methods

### 2.1 Network pharmacology analysis

#### 2.1.1 Screening of the active ingredients and targets of ZQCLD

The Traditional Chinese Medicine Systems Pharmacology Database and Analysis Platform (TCMSP) (https://tcmsp-e.com/tcmsp.php) [31] was used to screen the chemical components of ZQCLD (*Aurantii Fructus* and *Coptidis Rhizoma*) with the following criteria: oral bioavailability ≥30 % and drug-likeness ≥0.18 [32]. Targets for these active components were identified using the TCMSP database and standardised using the UniProt database (https://www.uniprot.org) [33].

#### 2.1.2 Identification of disease-related targets and disease–drug action targets

The GeneCards (http://www.genecards.org), OMIM (http://www.omim.org), and DisGeNET (http://www.disgenet.org) databases were used to search for disease-related targets, using the queries ‘food accumulation’, ‘food retention’, ‘dyspepsia’, and ‘fever’. Because the number of targets was large, targets in the GeneCards database with relevance scores higher than the median were chosen, and the operation was repeated once [34]. The targets obtained using the queries ‘food accumulation’, ‘food retention’, and ‘dyspepsia’ were summarised and deduplicated. The FAF-related targets were obtained by identifying the intersection of the above targets, and the targets were obtained using the query ‘fever’ using Venny 2.1.0 (https://bioinfogp.cnb.csic.es/tools/venny). Next, the intersection with the targets of ZQCLD was identified to obtain the disease-drug targets. Inflammation may be a key factor in FAF; therefore, the Gene Ontology (GO) functions of disease drug targets were analysed against the UniProt database. Disease-drug targets related to ‘inflammatory response’ were selected for further studies.

#### 2.1.3 Network construction and analysis

A protein-protein interaction (PPI) network was constructed using the STRING database (https://cn.string-db.org) with species limited to *Homo sapiens*. Core targets in the PPI network were selected using Cytoscape 3.8.0 [35] with the CytoNCA plugin [36]. Targets with scores above the median were selected for this study. The component–target network was constructed using Cytoscape 3.8.0, and the NetworkAnalyzer plugin was used to analyse the topology parameters.

#### 2.1.4 Function enrichment and pathway analysis

GO function enrichment analysis and Kyoto Encyclopedia of Genes and Genomes (KEGG) pathway enrichment analysis of the core targets were performed using R 4.4.2 with the R packages ‘clusterProfiler’ [37], ‘pathview’ [38], ‘org.Hs.eg.db’, and ‘ggplot2’. A threshold of *P* < 0.05 was set. GO enrichment analysis was conducted for the biological process (BP), molecular function (MF), and cellular component (CC) categories.

### 2.2 Animals and feed

Sixty 4-week-old SPF-grade healthy Sprague-Dawley rats (120 ± 10 g, 30 male and 30 female) were purchased from SiPeiFu Biotechnology Co., Ltd. (Beijing, China, licence number: SCXK (Beijing) 2019-0010). Rats were allowed to acclimate for 1 week before the experiments. All animals were housed in the animal laboratory of the Beijing University of Chinese Medicine.

Ordinary irradiated feed was provided by SiPeiFu Biotechnology Co., Ltd. (Beijing, China). Special high-calorie feed was prepared from rice crust, chocolate wafers, beef grains, and commercial wheat flour in a 1:2:2:1 ratio. The high-calorie feed had the same appearance and texture as ordinary feed. SiPeiFu Biotechnology Co., Ltd. (Beijing, China) was performed the procurement of raw materials, feed production, radiation sterilisation, and quality control.

### 2.3 Animal feeding and material collection

The 60 rats were divided into control, model, positive, low-, medium-, and high-dose groups according to a random number table, with five males and five females in each group. To avoid experimental bias owing to subjective human factors, the principle of blinding was followed. During the whole process of the animal experiment, the operator and recorder were unaware of the group and specifics of the experimental animals.

The experiment lasted for 14 days. During the experiment, all rats except those in the control group were given high-calorie feed and a special high-calorie suspension (Supplementary information 1) by gavage (1 mL per 100 g body weight) once a day. The rats in the control group were provided with basic feed and an equal volume of pure water. From days 11–14, rats in the positive control group were administered domperidone solution (0.315 mg/mL) (Supplementary information 1 and 2) by gavage (1 mL per 100 g body weight) once a day. Rats in the low-, medium-, and high-dose groups were administered ZQCLD solution (0.0475, 0.095, and 0.19 g/mL, respectively) (Supplementary information 1 and 2) by gavage (1 mL per 100 g body weight) once a day. The rats in the control and model groups were administered an equal volume of pure water.

On the morning of day 15, after overnight fasting with free access to water, each rat was administered 2 mL (2 g) of alimentary semisolid paste (Supplementary information 1) by gavage. After 30 min, the rats were anaesthetised by intraperitoneal injection of 20 % urethane (0.7 mL per 100 g body weight). Blood was collected from the abdominal aorta, held at room temperature for 4 h, and centrifuged at 1000×*g* for 20 min. The serum was stored at −80 °C. The upper and lower ends of the rat gastric body were knotted, and the gastric body and small intestine were removed at 4 °C. Brain tissue was removed and placed on ice. The hypothalamus was immediately harvested, weighed, fully homogenised, and centrifuged at 5000×*g* for 10 min at 4 °C. The supernatant was stored at −80 °C. After the measurement of the intestinal propulsion rate, 200 mg of jejunal tissue was collected, fully homogenised, and centrifuged at 5000×*g* for 10 min at 4 °C. The supernatant was stored at −80 °C.

### 2.4 Detection of signs

Prior to the experiment, the rectal temperatures of the rats in each group were recorded. Every morning, the rats were weighed and their daily food intake was recorded. The rectal temperature of the rats was measured. After material collection, the gastric emptying and intestinal propulsion rates were calculated (Supplementary Information 2). The levels of endotoxin in the serum, IL-1β and nNOS in the jejunum, and PGE2 and iNOS in the hypothalamus were measured using ELISA kits (Supplementary information 1).

### 2.5 Statistical analysis

Statistical analysis was performed using SPSS 22.0, and GraphPad Prism 9.0.0. The results are expressed as the mean ± standard deviation (SD) (X ± s). One-way ANOVA was conducted to compare data between groups, followed by LSD test. Differences were considered statistically significant at *P* < 0.05. Chi-squared test, correlation analysis, and principal component analysis (PCA) were performed using SPSS 22.0, and R 4.2.2. The R packages ‘ggstatsplot’, ‘factoextra’, ‘corrplot’, ‘ggplot2’, and ‘FactoMineR’ [39] were used.

## 3 Results

### 3.1 Active ingredients and targets of ZQCLD

Nineteen components were obtained from ZQCLD: five belonged to *Aurantii Fructus* and 14 belonged to *Coptidis Rhizoma*. A total of 16 compounds in ZQCLD had corresponding targets: five belonged to *Aurantii Fructus* and 11 belonged to *Coptidis Rhizoma* (Supplementary information 3, Table S1). After standardising the target names using the UniProt database and removing duplicates, 112 drug targets were obtained (Supplementary information 3, Table S2).

### 3.2 Disease-related targets and disease–drug action targets

A total of 1176 disease-related targets were identified after screening for deduplication and intersection. By intersecting the disease and drug targets, 67 disease–drug intersection targets were obtained, 20 of which were related to the ‘inflammatory response’ (Supplementary information 3, Table S3).

### 3.3 PPI network and component–target network analyses

**Fig. 1:**
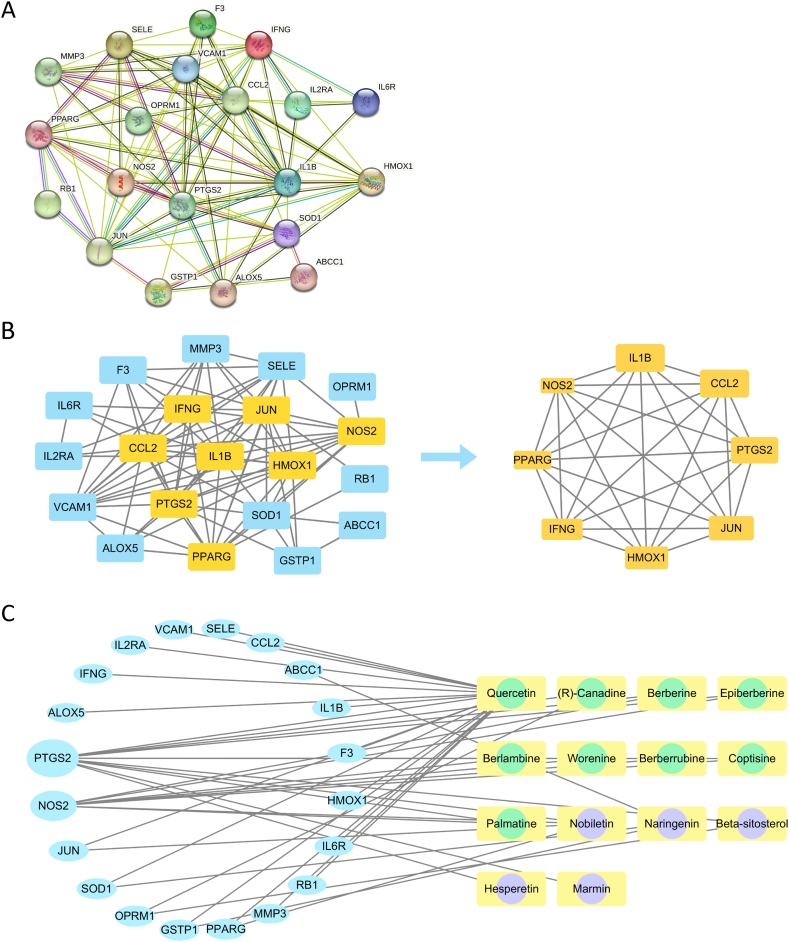
Network construction. **A** PPI network of disease-drug targets. **B** Selection of core targets. Yellow squares represent core targets. The size represents the degree of the relevant target. **C** Compound-target network. Light blue ellipses represent drug targets. The size represents the degree of the relevant target. Light yellow squares with green circles represent compounds from *Coptidis Rhizoma*. Light yellow squares with purple circles represent compounds from *Aurantii Fructus*. (For interpretation of the references to colour in this figure legend, the reader is referred to the Web version of this article.)

A PPI network of disease–drug action targets was constructed using the STRING database and Cytoscape 3.8.0. It contained 20 nodes and 89 edges, and the average node degree was 8.9 (Fig. 1A). The following core targets were obtained through the CytoNCA plugin: IL-1β, C–C motif chemokine 2 (CCL2), prostaglandin G/H synthase 2 (PTGS2), transcription factor AP-1 (JUN), haem oxygenase 1 (HMOX1), interferon-gamma (IFN-γ), peroxisome proliferator-activated receptor-gamma (PPAR-γ), and NOS2 (Fig. 1B). The component–target network was constructed using Cytoscape 3.8.0 (Fig. 1C). It contained 34 nodes (14 nodes belonging to the components and 20 nodes belonging to the targets) connected by 44 edges. The core components of ZQCLD, including quercetin (degree = 17), nobiletin (degree = 4), and naringenin (degree = 3), play important roles in this network, mainly by targeting PTGS2 and NOS2 (degree >2).

### 3.4 GO function and KEGG pathway enrichment analyses

**Fig. 2:**
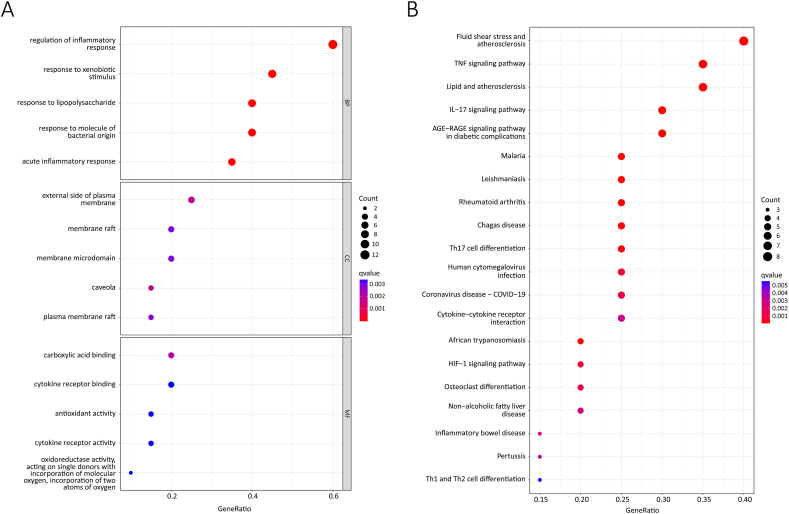
GO function enrichment and KEGG pathway enrichment analyses. **A** GO function bubble map (top 5 items in the BP, MF, and CC categories). **B** KEGG pathway bubble map (top 20 pathways). The colour of the dot represents the q-value, and the size of the dot represents the number of core targets mapped to the relevant GO term or KEGG pathway. (For interpretation of the references to colour in this figure legend, the reader is referred to the Web version of this article.)

According to our GO function enrichment results, the core targets of ZQCLD in the BP category were enriched in 1091 items, including regulation of the inflammatory response, acute inflammatory response, response to xenobiotic stimulus, response to lipopolysaccharide, and response to molecules of bacterial origin. In the CC category, the core targets were enriched in 19 items, including the caveola, external side of the plasma membrane, plasma membrane raft, membrane raft, and membrane microdomain. In the MF category, the core targets were enriched in 74 items, including carboxylic acid binding, antioxidant activity, cytokine receptor activity, cytokine receptor binding, and oxidoreductase activity (Supplementary information 3, Table S4). The top five enrichment results for the BP, CC, and MF categories are shown in Fig. 2A.

KEGG pathway enrichment analysis revealed that the core targets of ZQCLD were enriched in 50 pathways, including fluid shear stress and atherosclerosis, TNF signalling pathway, IL-17 signalling pathway, advanced glycation end product (AGE)-receptor for AGEs (RAGE) signalling pathway in diabetic complications, and malaria (Supplementary information 3, Table S5). The top 20 enrichment results are shown in Fig. 2B.

### 3.5 Body weight and food intake

**Fig. 3:**
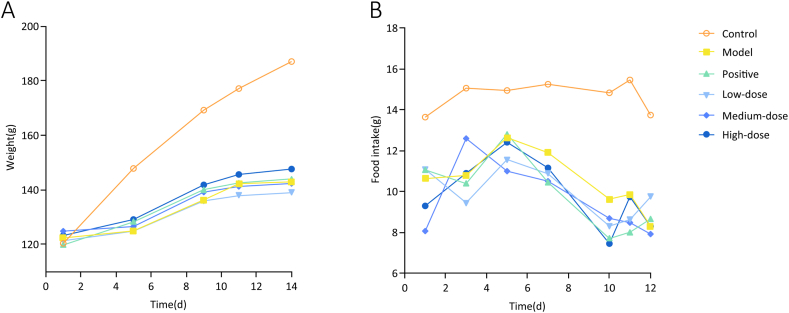
Effect of ZQCLD on body weight and food intake. **A** Average body weight of rats in each group. **B** Average daily food intake of rats in each group.

**Fig. 4:**
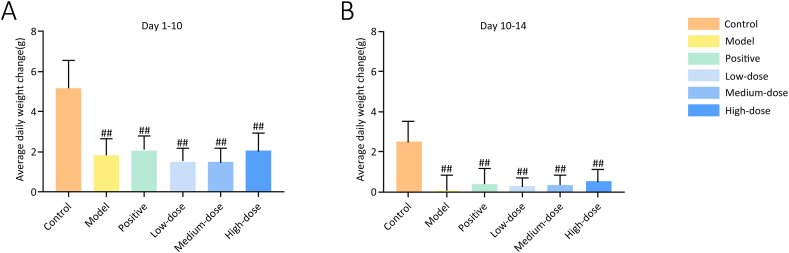
Effect of ZQCLD on average daily weight change. **A** Average daily weight change before the treatment. **B** Average daily weight change during the treatment.

High-calorie diets can induce marked alterations in the body weight and food intake. Before the experiment, there was no significant difference in body weight between the groups. However, during the experiment, the body weight and food intake of rats in the model group and each treatment group were significantly lower than those in the control group (Fig. 3). The daily change in rat body weight in the model and treatment groups was significantly lower than that in the control group (*P* < 0.01) (Fig. 4).

### 3.6 Body temperature

**Fig. 5:**
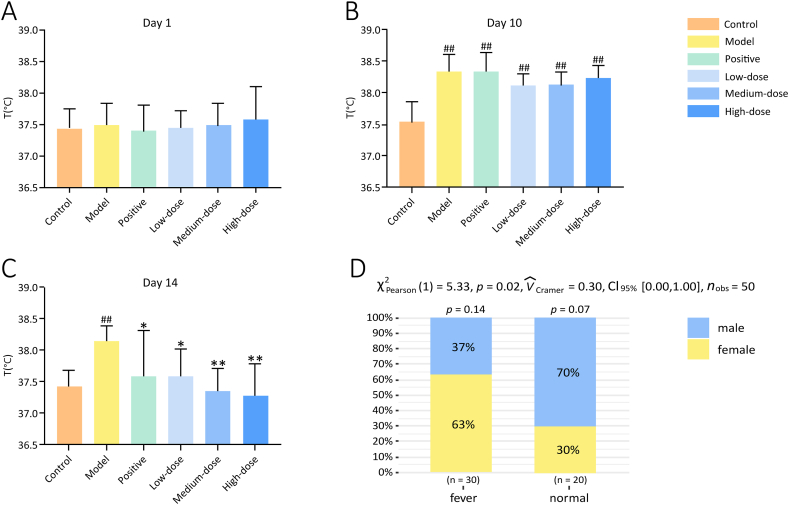
Effect of ZQCLD on body temperature. **A** Body temperature before the experiment. **B** Body temperature before the treatment. **C** Body temperature after the treatment. **D** Chi-squared test of gender and body temperature on day 10 (before treatment). ^#^*P* < 0.05, ^##^*P* < 0.01 vs. the control group; *P < 0.05, **P < 0.01 vs. the model group (*n* = 6).

Analysis of the body temperature of the rats in each group before the experiment revealed that the 95 % confidence interval of the body temperature range was 37.58 °C–37.84 °C. Therefore, the data of control rats with a body temperature below 37.58 °C and model rats with a body temperature above 37.84 °C were included in the subsequent data analysis (six rats in each group, three males and three females in the control group). Based on the results of the chi-square test (Fig. 5D), changes in body temperature after administration of the special high-calorie diet varied by sex. Of the 50 FA rats, 30 developed fever, and 20 did not. Of the rats that developed fever, 63 % were female and 37 % were male (3 males and 3 females in the model group, 2 males and 4 females in the positive group, 2 males and 4 females in the low-dose group, 2 males and 4 females in the medium-dose group, and 2 males and 4 females in the high-dose group); of the rats that did not develop fever, 70 % were male, and 30 % were female. A considerably greater percentage of female rats developed fever while on a special high-calorie diet than male rats (*P* < 0.05).

Before the experiment, there was no difference in the temperature between the rats in each group (Fig. 5A). Before treatment (day 10), the body temperatures of the rats in the model group and each treatment group were significantly elevated (*P* < 0.01) (Fig. 5B). On the last day of the experiment (day 14), the body temperature of rats in the model group remained noticeably higher than that of rats in the control group (*P* < 0.01). The body temperature of the rats in each treatment group decreased to different degrees compared to that in the model group (*P* < 0.05) (Fig. 5C).

### 3.7 Gastric emptying rate and intestinal propulsion rate

**Fig. 6:**
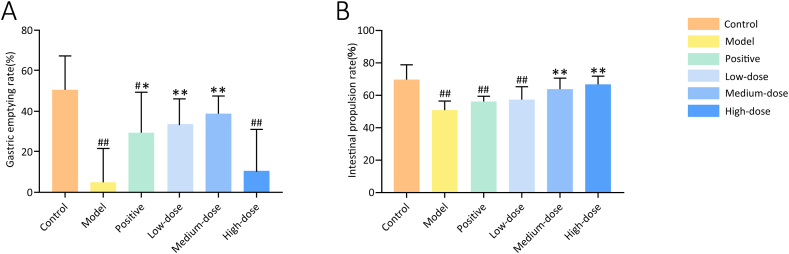
Effect of ZQCLD on the gastric emptying rate and intestinal propulsion rate. **A** Gastric emptying rate. **B** Intestinal propulsion rate. ^#^*P* < 0.05, ^##^*P* < 0.01 vs. the control group; *P < 0.05, **P < 0.01 vs. the model group (*n* = 6).

To investigate the gastrointestinal motor function of rats in each group, gastric emptying and intestinal propulsion rates were measured. Gastric emptying and intestinal propulsion rates in the model group were significantly lower than those in the control group (*P* < 0.01). Compared with the model group, the gastric emptying rate significantly increased in the positive and low- and medium-dose groups (*P* < 0.05), and the intestinal propulsion rate significantly increased in the medium- and high-dose groups (*P* < 0.01) (Fig. 6).

### 3.8 ELISA results

**Fig. 7:**
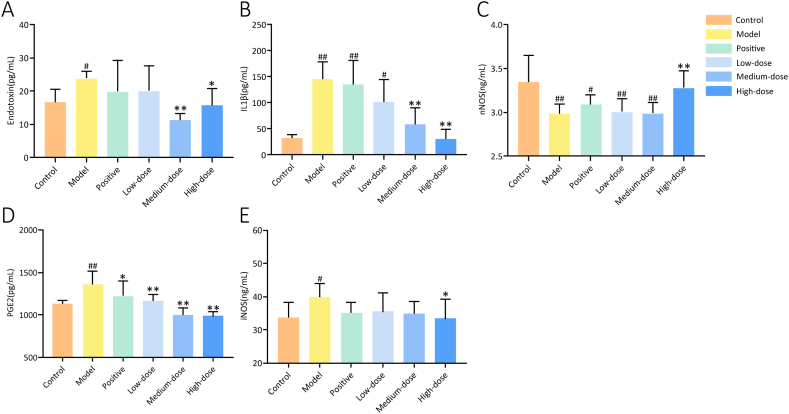
ELISA results of serum endotoxin, jejunal IL-1β and nNOS, and hypothalamic PGE2 and iNOS levels. **A** Endotoxin. **B** IL-1β. **C** nNOS. **D** PGE2. **E** iNOS. ^#^*P* < 0.05, ^##^*P* < 0.01 vs. the control group; *P < 0.05, **P < 0.01 vs. the model group (*n* = 6).

Compared with those in the control group, the nNOS level in the jejunum decreased in the model group, whereas the serum endotoxin, jejunal IL-1β, and hypothalamic PGE2 and iNOS levels increased to different degrees (*P* < 0.05) (Fig. 7). Compared with those in the model group, the hypothalamic PGE2 levels (Fig. 7D) decreased to different degrees (*P* < 0.05) in all treatment groups; serum endotoxin (Fig. 7A) and jejunal IL-1β levels (Fig. 7B) decreased to different degrees in the medium- and high-dose groups (*P* < 0.05); the jejunal nNOS level (Fig. 7C) was elevated in the high-dose group, whereas the hypothalamic iNOS level (Fig. 7E) was reduced (*P* < 0.05).

### 3.9 Correlation analysis and principal component analysis

**Fig. 8:**
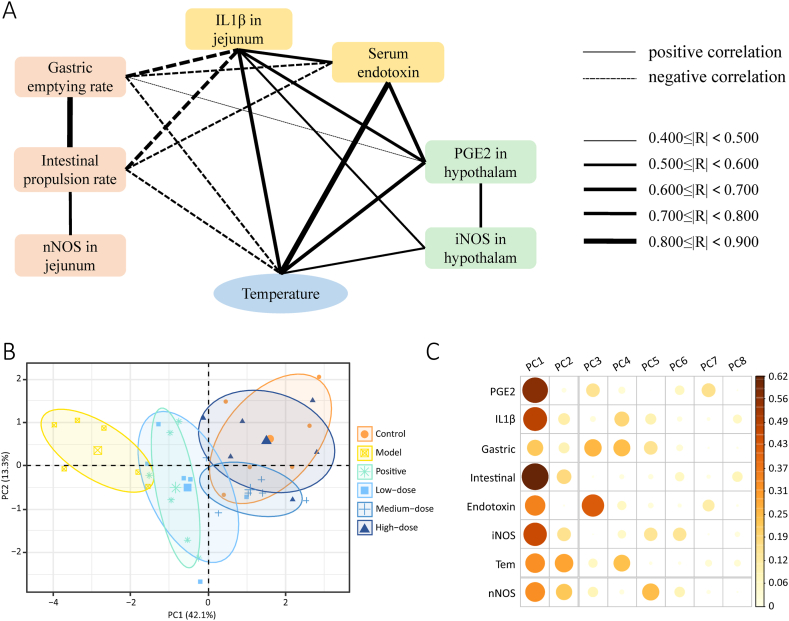
Correlation analysis and principal component analysis results. **A** Correlation analysis (only significant results are presented). **B** Principal component analysis. **C** Contribution of variables to each PC. The colour and size of the dots represent the contribution degree of the variables. (For interpretation of the references to colour in this figure legend, the reader is referred to the Web version of this article.)

To further explore the presence of a correlation between the indicators, Pearson correlation analysis was performed (Fig. 8A). The closer the |R| value is to 1, the greater the correlation. Among all treatment groups, the medium-dose group showed the best overall effect and the most stable data and was therefore included in the analysis together with the control and model groups. The gastric emptying rate and intestinal propulsion rate were negatively correlated with body temperature, whereas jejunal IL-1β, serum endotoxin, and hypothalamic PGE2 and iNOS levels were positively correlated with body temperature, with the highest |R| value for jejunal IL-1β. Among the three indicators of gastrointestinal motility, intestinal propulsion rate was positively correlated with gastric emptying rate and jejunal nNOS levels. Both the gastric emptying rate and intestinal propulsion rate were negatively correlated with jejunal IL-1β and serum endotoxin levels, which are both related to inflammation. Furthermore, gastric emptying and intestinal propulsion rates were negatively correlated with hypothalamic PGE2 and iNOS levels, both of which are related to thermoregulation. A positive correlation between hypothalamic PGE2 and iNOS levels was observed. There was a positive correlation between jejunal IL-1β and serum endotoxin levels, which were both positively correlated with hypothalamic PGE2 levels. In addition, jejunal IL-1β was positively correlated with hypothalamic nNOS.

PCA was performed to assess the overall pattern and differences between the groups. Principal components 1 (PC1) and PC2 explained 55.4 % of the total variance (Fig. 8B). The intestinal propulsion rate and PGE2, IL-1β, and iNOS levels were the main contributors to PC1, and temperature was the main contributor to PC2 (Fig. 8C). The control and model groups were separated from each other, whereas the control and high-dose groups were closer and had an overlap, suggesting that the overall condition of the high-dose group was closest to that of the control group, and the treatment effect was greatest in the high-dose group, followed by the medium-dose, low-dose, and positive control groups.

## 4 Discussion

In this study, network pharmacology and animal experiments were conducted to reveal the specific mechanisms underlying fever caused by FA and the action of ZQCLD. The results showed that the main target proteins of ZQCLD in the treatment of FAF were IL-1β, CCL2, PTGS2, JUN, HMOX1, IFN-γ, PPAR-γ, and NOS2. IL-1β is a typical potent pro-inflammatory cytokine that also plays an important role in the intestinal inflammatory response [40,41]. As a major endogenous pyrogen, it induces prostaglandin synthesis and causes fever [42]. A recent study showed that IL-1β is also involved in intestinal damage in largemouth bass caused by a high-carbohydrate diet, which can be alleviated by berberine, one of the active components of *Coptidis Rhizoma* [43]. PTGS2 is a dual cyclooxygenase and peroxidase that is involved in prostanoid biosynthesis [44]. PGE2, one of the most important products of PTGS2, is an important central heat mediator that is activated by IL-1β and binds to its receptor, stimulating the release of cyclic adenosine monophosphate (cAMP), which ultimately mediates fever [45,46]. NOS2 is nitric oxide synthase, which is a key rate-limiting enzyme in NO synthesis. There are three types of NO synthases: nNOS/NOS1, iNOS/NOS2, and endothelial NO synthases (eNOS, NOS3) [47]. iNOS is involved in inflammation and enhances the synthesis of pro-inflammatory mediators [48]. iNOS is closely associated with thermoregulation because it catalyzes the production of NO as a central heating medium, acting on the thermoregulatory centre and mediating an increase in body temperature [49]. nNOS is primarily expressed in the nervous system. In the digestive system, nNOS^+^ neurones relax the smooth muscles and inhibit gastrointestinal motility by producing and releasing NO [50,51].

Based on our network pharmacology results, IL-1β and iNOS were selected as experimental indicators. As an important product of PTSG2, PGE2 plays a key role in thermoregulation and was therefore included in the assay. Because nNOS belongs to the NOS family and plays an important role in regulating gastrointestinal motility, it was included in the assay to further investigate the regulatory mechanism of gastrointestinal motility in FA. Although PGE2 and nNOS did not appear in our network pharmacology results, they were included in follow-up experiments to ensure the comprehensiveness, depth, and integrity of the study.

The TCM theory holds that children should not be overfed. Modern medicine also provides evidence that excessive eating during childhood can harm the future or even lifelong eating patterns and metabolic status [9,10]. However, children are often prone to food overconsumption because of the rising standard of living and excessive parental concerns over paediatric nutritional deficiencies [1,52]. Such eating habits can contribute to the development of FA, or even FAF. Without intervention, children's growth and development may be further affected. TCM intervention can improve FA and damage in children caused by overeating.

Based on the above understanding of FA in TCM and the current dietary patterns in children, the present study optimised an animal model based on previous studies and adopted a cafeteria diet (CFA) [53,54] for feeding rats. CFA consists of delicious but less healthy foods similar to those consumed by humans (such as hot dogs, cakes, chocolate wafers, and rice crust). Compared to conventional high-calorie, high-fat, and similar diets, CFA has a richer taste, smell, and texture, which better mimics the unhealthy foods consumed by humans. Through the CFA and ‘free diet + forced diet’ feeding modes, the animal model of this study better mimicked the aetiology and pathogenesis of clinical paediatric FAF. In addition, based on the experimental results, changes in the indices of the rats in the model group were consistent with the disease targets predicted by network pharmacology. This verified the ability of this model to replicate the characteristics of FAF at the molecular level to a certain extent.

Following an excessively high-calorie diet for 14 days, the growth of the model animals was significantly slower than that of the normal group. Moreover, food intake, gastric emptying rate, and intestinal propulsion rate decreased, and the stool became dry or did not form. These results indicated that FA occurred, gastrointestinal function was altered, and the growth and development of 4-week-old rats were affected. Based on the ELISA results, jejunal IL-1β and serum endotoxin levels in the model rats increased, which indicated that the high-calorie diet led to local inflammatory responses in the intestine, affecting the intestinal barrier, through which endotoxin can enter the systemic circulation. Caesar et al. [55] reported elevated serum endotoxin levels without bacterial translocation in mice fed a high-fat diet for 11 weeks. This indicates that rather than the gut microbiota moving from the intestine, molecules that promote inflammation are responsible for the increase in serum endotoxin levels in this process. The endotoxin entering the systemic circulation activated Toll-like receptor 4 (TLR4) in the body, promoted the synthesis of inflammatory cytokines, such as IL-1, tumour necrosis factor-alpha (TNF-α), and IL-6, and caused a systemic inflammatory response [56,57]. The increase in serum endotoxin levels in model rats also indicated the development of a systemic inflammatory response. The endotoxin and inflammatory cytokines in rats further stimulated the related pathways and receptors, ultimately leading to increased levels of PGE2 and iNOS in the hypothalamus [49,58,59], which mediated the increase in body temperature and the development of FAF in rats.

According to our correlation analysis, the gastric emptying rate and intestinal propulsion rate, which reflect gastrointestinal motility, were both negatively correlated with jejunal IL-1β, serum endotoxin, hypothalamic PGE2 and iNOS levels, and body temperature. In contrast, jejunal IL-1β, which reflects the state of intestinal inflammation, was positively correlated with serum endotoxin, hypothalamic PGE2 and iNOS levels, and body temperature. The results indicated that gastrointestinal motility, intestinal inflammation, and fever in rats were correlated and subject to mutual influence, rather than presenting three independent aspects, suggesting that FAF cannot be regarded as a simple combination of FA and fever.

In general, nNOS plays a negative regulatory role in gastrointestinal smooth muscle movement. The nNOS + neurones relax smooth muscles and inhibit gastrointestinal motility by promoting NO release [50,51]. However, the loss or reduction of nNOS^+^ neurones may also cause gastrointestinal motility dysfunction [60], which has been reported in a range of human enteric neuropathies, such as slow transit constipation [61] and gastroparesis [62]. Moreover, nNOS-deficient mice have been shown to exhibit several gastrointestinal motility disorders, including delayed gastric emptying [63] and slow colonic transit [64]. In our experiment, the decrease in nNOS in the model group was accompanied by a decrease in the gastric emptying and intestinal propulsion rates, suggesting that the decrease in the gastric emptying and intestinal propulsion rates in the model rats may be due to a reduction in nNOS rather than an increase in NO produced by nNOS.

Based on our network pharmacology results, 14 active ingredients of ZQCLD in the treatment of FAF were identified, five of which belonged to *Aurantii Fructus* (nobiletin, naringenin, beta-sitosterol, hesperetin, and marmin) and nine of which belonged to *Coptidis Rhizoma* (quercetin, (R)-canadine, berberine, epiberberine, berlambine, worenine, berberrubine, coptisine, and palmatine). A previous study has shown that *Aurantii Fructus* promoted gastrointestinal motility in an FD rat model [65]. An ethanol extract of *Aurantii Fructus* ameliorated intestinal inflammation and regulates the intestinal barrier in FD model rats [66]. *Coptidis Rhizoma* has anti-inflammatory [67,68] and anti-pyretic effects [69]. Berberine has shown therapeutic effects in both ulcerative colitis model rats [24] and diabetic model mice [67] by exerting anti-inflammatory effects and regulating the gut microbiome. These results indicate that ZQCLD can alleviate high-calorie diet-induced FAF. Combined with our PCA results, the findings indicate that the high-dose ZQCLD group experienced the greatest overall effect; the body temperature, intestinal propulsion rate, serum endotoxin, jejunal IL-1β and nNOS, and hypothalamic PGE2 and iNOS levels were all restored to normal levels. In contrast, medium-dose ZQCLD had the most consistent effect, with the most concentrated points, smallest circle in the PCA plot, and the lowest SD value of each index, which is why the medium-dose group was included in the correlation analysis together with the control and model groups. After administration of ZQCLD or domperidone, the growth and food intake of the model rats did not significantly improve. This may be related to shorter administration times. At the same time, these findings suggest that a short-term excessively high-calorie diet caused significant damage to young rats, and even short-term administration did not completely restore their health.

The conventional prokinetic drug domperidone, which was used as the positive control drug [70,71], can improve the gastric emptying rate, body temperature, and hypothalamic PGE2 levels in FAF rats, suggesting that the treatment of dyspepsia helps reduce body temperature to some extent. However, it had no significant beneficial effects on other indices, and its overall effect was inferior to that of medium- and high-dose ZQCLD, according to our PCA results.

## 5 Conclusion

The present study suggests that ZQCLD can reduce intestinal inflammation, restore gastrointestinal motility, and improve high-calorie diet-induced FAF by regulating the levels of IL-1β and nNOS in the jejunum, endotoxin in the serum, and PGE2 and iNOS in the hypothalamus. The present study not only improves our understanding of the active ingredients and molecular mechanisms of ZQCLD, but also provides a reference for the clinical treatment of FAF and application of ZQCLD. However, the mechanism by which ZQCLD affects high-calorie diet-induced FAF requires further exploration.

## Ethics statement

All protocols in this study were performed in accordance with the National Institutes of Health – Office of Laboratory Animal Welfare policies and laws and in compliance with the Animal Research: Reporting of In Vivo Experiments (ARRIVE) guidelines. The animal experiment was approved by the Animal Ethics Committee of Beijing University of Chinese Medicine (license number: BUCM-4-2022061505-2075).

## Data availability statement

The datasets used or analysed during the current study are available from the corresponding author upon reasonable request.

## Funding

This study was supported by Municipal Innovation and Entrepreneurship Training Program for College Students (S202210026011) and the Scientific Research Project for Students of Beijing University of Chinese Medicine (XBB23064).

## CRediT authorship contribution statement

**Chuxin Zhang:** Writing – original draft, Project administration, Methodology, Formal analysis, Conceptualization. **Ruoshi Zhang:** Methodology, Investigation, Data curation. **Yuli Cheng:** Visualization, Software. **Jingpeng Chen:** Investigation. **Ruizi Zhu:** Investigation. **Lin Gao:** Writing – review & editing, Supervision, Resources. **Mei Han:** Writing – review & editing, Supervision, Methodology.

## Declaration of competing interest

The authors declare that they have no known competing financial interests or personal relationships that could have appeared to influence the work reported in this paper.

## Abbreviations

FA: Food accumulation
TCM: Traditional Chinese medicine
FD: Functional dyspepsia
FAF: Food accumulation fever
ZQCLD: *Zhiqiao Chuanlian* Decoction
OB: Oral bioavailability
DL: Drug likeness
ELISA: Enzyme-linked immunosorbent assay
IL-1β: Interleukin-1β
PGE2: Prostaglandin E2
iNOS/NOS2: Inducible nitric oxide synthase
nNOS/NOS1: Neuronal nitric oxide synthase
eNOS/NOS3: Endothelial nitric oxide synthase
PPI: Protein-protein interaction
GO: Gene Ontology
KEGG: Kyoto Encyclopedia of Genes and Genomes
BP: Biological process
MF: Molecular function
CC: Cellular component
PBS: Phosphate-buffered saline
SD: Standard deviation
CCL2: C–C motif chemokine 2
PTGS2: Prostaglandin G/H synthase 2
JUN: Transcription factor AP-1
HMOX1: Haem oxygenase 1
IFN-γ: Interferon gamma
PPAR-γ: Peroxisome proliferator-activated receptor gamma
AGE: Advanced glycation end product
RAGE: Receptor for AGEs
PCA: Principal component analysis
PC: Principal component
cAMP: Cyclic adenosine monophosphate
CFA: Cafeteria diet
TLR4: Toll-like receptor 4
TNF-α: Tumour necrosis factor-alpha
PI3K: Phosphatidylinositol 3-kinase
Akt: Threonine-protein kinase
