# Supplementary material for: Role of Zhiqiao Chuanlian decoction in the treatment of food accumulation fever: Network pharmacology and animal experiments
Source: Heliyon. 2024 Apr 17;10(8):e29813. doi: 10.1016/j.heliyon.2024.e29813 (PMC11053291; doi:10.1016/j.heliyon.2024.e29813)
Supplement: Multimedia component 1 [file mmc1.docx]

**Supplementary information 1**

**Method**

**1. Reagent preparation**

***1.1. Special high-calorie suspension***

The special high-calorie suspension was prepared by fully pulverising special feed and then suspending 60 g of special feed powder in 240 mL of distilled water.

***1.2. Domperidone solution***

Domperidone tablets were completely pulverised, and the powder was dissolved in pure water. Rats received 0.315 mg/mL per 100 g body weight at room temperature (the human clinical equivalent dose), based on the conversion ratio of surface area between rats and humans (6.3).

***1.3. ZQCLD solution***

*Aurantii Fructus* and *Coptidis Rhizoma* granules were mixed at a ratio of 2:1 and dissolved in pure water. Rats in the low-, medium-, and high-dose groups received 0.0475, 0.095, and 0.19 g/mL per 100 g body weight (0.5, 1, and 2 times the human clinical equivalent dose), respectively, based on the conversion ratio of surface area between rats and humans (6.3).

***1.4. Alimentary semi-solid paste***

5 g of carboxymethyl cellulose sodium was dissolved in 125 mL of pure water, and 8 g of milk powder, 4 g of sugar, 4 g of starch, and 1.5 g of activated carbon powder were added. Finally, the mixture was a black semi-solid paste with a volume of approximately 150 mL and a weight of approximately 150 g.

**2. Gastric emptying rate and intestinal propulsion rate**

The stomach was taken, the surface moisture was removed using absorbent paper, and the stomach was weighed (total gastric weight). Then the stomach was cut open along the greater curvature of the stomach with scissors and washed with physiological saline, moisture was removed with absorbent paper, and the net gastric weight was recorded. The gastric emptying rate was calculated as follows: gastric emptying rate = [(total gastric weight − net gastric weight) / weight of alimentary paste] × 100%.

Then the small intestine of rats was straightened, and the total length from the pylorus to the ileocecal junction (Length_P-I_) and the distance from the pylorus to the front end of the black paste (Length_P-B_) were measured. The intestinal propulsion rate was calculated as follows: intestinal propulsion rate = (Length_P-B_ / Length_P-I_) × 100%.
