# Supplementary material for: Role of Zhiqiao Chuanlian decoction in the treatment of food accumulation fever: Network pharmacology and animal experiments
Source: Heliyon. 2024 Apr 17;10(8):e29813. doi: 10.1016/j.heliyon.2024.e29813 (PMC11053291; doi:10.1016/j.heliyon.2024.e29813)
Supplement: Multimedia component 2 [file mmc2.docx]

**Supplementary information 2**

**Equipment and reagents**

The following material was used:

Rat endotoxin enzyme-linked immunosorbent assay (ELISA) kit (Shanghai Jianglai Biotechnology Co., Ltd., China, JL13330).

Rat interleukin-1β (IL-1β) ELISA kit (Shanghai Jianglai Biotechnology Co., Ltd., China, JL20884).

Rat prostaglandin E2 (PGE2) ELISA kit (Shanghai Jianglai Biotechnology Co., Ltd., China, JL20884)

Rat inducible nitric oxide synthase (iNOS, NOS2) ELISA kit (Shanghai Enzyme-linked Biotechnology Co., Ltd., China, ml1003127-J)

Rat neuronal nitric oxide synthase (nNOS, NOS1) ELISA kit (Shanghai Enzyme-linked Biotechnology Co., Ltd., China, ml123696-J).

Carboxymethyl cellulose sodium (Shanghai Yuanye Biotechnology Co., Ltd., China, S14017).

Urethane (Shanghai Yuanye Biotechnology Co., Ltd., China, S11036).

Stir-fried *Aurantii Fructus* and *Coptidis Rhizoma* granules (Guangdong Efong Pharmaceutical Co., Ltd., China, No. 1101913, No. 1106923).

Domperidone tablets were obtained from Xi'an Janssen Pharmaceutical Co., Ltd. (China, No. H10910003).
