# Supplementary material for: Role of Zhiqiao Chuanlian decoction in the treatment of food accumulation fever: Network pharmacology and animal experiments
Source: Heliyon. 2024 Apr 17;10(8):e29813. doi: 10.1016/j.heliyon.2024.e29813 (PMC11053291; doi:10.1016/j.heliyon.2024.e29813)
Supplement: Multimedia component 3 [file mmc3.docx]

**Supplementary information 3**

Table S1 Information of active components of Zhiqiao Chuanglian Dang

| Mol ID | Molecule Name | MW | OB (%) | DL | Drug |
| --- | --- | --- | --- | --- | --- |
| MOL000358 | Beta-sitosterol | 414.79 | 36.91 | 0.75 | Aurantii Fructus |
| MOL002341 | Hesperetin | 302.3 | 70.31 | 0.27 | Aurantii Fructus |
| MOL004328 | Naringenin | 272.27 | 59.29 | 0.21 | Aurantii Fructus |
| MOL005828 | Nobiletin | 402.43 | 61.67 | 0.52 | Aurantii Fructus |
| MOL013381 | Marmin | 332.43 | 38.23 | 0.31 | Aurantii Fructus |
| MOL000098 | Quercetin | 302.25 | 46.43 | 0.28 | Coptidis Rhizoma |
| MOL000622 | Magnograndiolide | 266.37 | 63.71 | 0.19 | Coptidis Rhizoma |
| MOL000762 | Palmidin A | 510.52 | 35.36 | 0.65 | Coptidis Rhizoma |
| MOL000785 | Palmatine | 352.44 | 64.6 | 0.65 | Coptidis Rhizoma |
| MOL001454 | Berberine | 336.39 | 36.86 | 0.78 | Coptidis Rhizoma |
| MOL001458 | Coptisine | 320.34 | 30.67 | 0.86 | Coptidis Rhizoma |
| MOL002668 | Worenine | 334.37 | 45.83 | 0.87 | Coptidis Rhizoma |
| MOL002894 | Berberrubine | 322.36 | 35.74 | 0.73 | Coptidis Rhizoma |
| MOL002897 | Epiberberine | 336.39 | 43.09 | 0.78 | Coptidis Rhizoma |
| MOL002903 | (R)-Canadine | 339.42 | 55.37 | 0.77 | Coptidis Rhizoma |
| MOL002904 | Berlambine | 351.38 | 36.68 | 0.82 | Coptidis Rhizoma |
| MOL002907 | Corchoroside A_qt | 404.55 | 104.95 | 0.78 | Coptidis Rhizoma |
| MOL008647 | Moupinamide | 313.38 | 86.71 | 0.26 | Coptidis Rhizoma |
| MOL013352 | Obacunone | 454.56 | 43.29 | 0.77 | Coptidis Rhizoma |

OB: oral bioavailability; DL: drug likeness

Table S2 Information of Targets of Zhiqiao Chuanglian Dang

| Target Name | Mol ID | Mol Name | Drug |
| --- | --- | --- | --- |
| PGR | MOL000358 | Beta-sitosterol | Aurantii Fructus |
| NCOA2 | MOL000358 | Beta-sitosterol | Aurantii Fructus |
| PTGS1 | MOL000358 | Beta-sitosterol | Aurantii Fructus |
| PTGS2 | MOL000358 | Beta-sitosterol | Aurantii Fructus |
| HSP90AB1 | MOL000358 | Beta-sitosterol | Aurantii Fructus |
| KCNH2 | MOL000358 | Beta-sitosterol | Aurantii Fructus |
| DRD1 | MOL000358 | Beta-sitosterol | Aurantii Fructus |
| CHRM3 | MOL000358 | Beta-sitosterol | Aurantii Fructus |
| CHRM1 | MOL000358 | Beta-sitosterol | Aurantii Fructus |
| SCN5A | MOL000358 | Beta-sitosterol | Aurantii Fructus |
| CHRM4 | MOL000358 | Beta-sitosterol | Aurantii Fructus |
| ADRA1A | MOL000358 | Beta-sitosterol | Aurantii Fructus |
| CHRM2 | MOL000358 | Beta-sitosterol | Aurantii Fructus |
| ADRA1B | MOL000358 | Beta-sitosterol | Aurantii Fructus |
| ADRB2 | MOL000358 | Beta-sitosterol | Aurantii Fructus |
| CHRNA2 | MOL000358 | Beta-sitosterol | Aurantii Fructus |
| SLC6A4 | MOL000358 | Beta-sitosterol | Aurantii Fructus |
| OPRM1 | MOL000358 | Beta-sitosterol | Aurantii Fructus |
| GABRA1 | MOL000358 | Beta-sitosterol | Aurantii Fructus |
| BCL2 | MOL000358 | Beta-sitosterol | Aurantii Fructus |
| MAP2 | MOL000358 | Beta-sitosterol | Aurantii Fructus |
| PTGS1 | MOL002341 | Hesperetin | Aurantii Fructus |
| SCN5A | MOL002341 | Hesperetin | Aurantii Fructus |
| PTGS2 | MOL002341 | Hesperetin | Aurantii Fructus |
| HSP90AB1 | MOL002341 | Hesperetin | Aurantii Fructus |
| NCOA2 | MOL002341 | Hesperetin | Aurantii Fructus |
| NCOA1 | MOL002341 | Hesperetin | Aurantii Fructus |
| CAMKMT | MOL002341 | Hesperetin | Aurantii Fructus |
| DPEP1 | MOL004328 | Naringenin | Aurantii Fructus |
| BCL2 | MOL004328 | Naringenin | Aurantii Fructus |
| MAPK3 | MOL004328 | Naringenin | Aurantii Fructus |
| MAPK1 | MOL004328 | Naringenin | Aurantii Fructus |
| FASN | MOL004328 | Naringenin | Aurantii Fructus |
| LDLR | MOL004328 | Naringenin | Aurantii Fructus |
| SOD1 | MOL004328 | Naringenin | Aurantii Fructus |
| CAT | MOL004328 | Naringenin | Aurantii Fructus |
| HMGCR | MOL004328 | Naringenin | Aurantii Fructus |
| GSTP1 | MOL004328 | Naringenin | Aurantii Fructus |
| GSR | MOL004328 | Naringenin | Aurantii Fructus |
| ABCC1 | MOL004328 | Naringenin | Aurantii Fructus |
| AKR1C1 | MOL004328 | Naringenin | Aurantii Fructus |
| GOT1 | MOL004328 | Naringenin | Aurantii Fructus |
| ABAT | MOL004328 | Naringenin | Aurantii Fructus |
| SOAT1 | MOL004328 | Naringenin | Aurantii Fructus |
| NOS2 | MOL005828 | Nobiletin | Aurantii Fructus |
| PTGS1 | MOL005828 | Nobiletin | Aurantii Fructus |
| KCNH2 | MOL005828 | Nobiletin | Aurantii Fructus |
| ESR1 | MOL005828 | Nobiletin | Aurantii Fructus |
| AR | MOL005828 | Nobiletin | Aurantii Fructus |
| PPARG | MOL005828 | Nobiletin | Aurantii Fructus |
| PTGS2 | MOL005828 | Nobiletin | Aurantii Fructus |
| F7 | MOL005828 | Nobiletin | Aurantii Fructus |
| ESR2 | MOL005828 | Nobiletin | Aurantii Fructus |
| DPP4 | MOL005828 | Nobiletin | Aurantii Fructus |
| HSP90AB1 | MOL005828 | Nobiletin | Aurantii Fructus |
| CHEK1 | MOL005828 | Nobiletin | Aurantii Fructus |
| PRSS1 | MOL005828 | Nobiletin | Aurantii Fructus |
| NCOA2 | MOL005828 | Nobiletin | Aurantii Fructus |
| CAMKMT | MOL005828 | Nobiletin | Aurantii Fructus |
| GSK3B | MOL005828 | Nobiletin | Aurantii Fructus |
| SCN5A | MOL005828 | Nobiletin | Aurantii Fructus |
| BCL2 | MOL005828 | Nobiletin | Aurantii Fructus |
| JUN | MOL005828 | Nobiletin | Aurantii Fructus |
| TP53 | MOL005828 | Nobiletin | Aurantii Fructus |
| MAPK8 | MOL005828 | Nobiletin | Aurantii Fructus |
| PLA2G4A | MOL005828 | Nobiletin | Aurantii Fructus |
| PTGS2 | MOL013381 | Marmin | Aurantii Fructus |
| CA2 | MOL013381 | Marmin | Aurantii Fructus |
| ADRB2 | MOL013381 | Marmin | Aurantii Fructus |
| PTGS1 | MOL000098 | Quercetin | Coptidis Rhizoma |
| AR | MOL000098 | Quercetin | Coptidis Rhizoma |
| PPARG | MOL000098 | Quercetin | Coptidis Rhizoma |
| PTGS2 | MOL000098 | Quercetin | Coptidis Rhizoma |
| HSP90AB1 | MOL000098 | Quercetin | Coptidis Rhizoma |
| NCOA2 | MOL000098 | Quercetin | Coptidis Rhizoma |
| DPP4 | MOL000098 | Quercetin | Coptidis Rhizoma |
| AKR1B1 | MOL000098 | Quercetin | Coptidis Rhizoma |
| PRSS1 | MOL000098 | Quercetin | Coptidis Rhizoma |
| KCNH2 | MOL000098 | Quercetin | Coptidis Rhizoma |
| SCN5A | MOL000098 | Quercetin | Coptidis Rhizoma |
| ADRB2 | MOL000098 | Quercetin | Coptidis Rhizoma |
| MMP3 | MOL000098 | Quercetin | Coptidis Rhizoma |
| F7 | MOL000098 | Quercetin | Coptidis Rhizoma |
| RXRA | MOL000098 | Quercetin | Coptidis Rhizoma |
| ACHE | MOL000098 | Quercetin | Coptidis Rhizoma |
| GABRA1 | MOL000098 | Quercetin | Coptidis Rhizoma |
| MAOB | MOL000098 | Quercetin | Coptidis Rhizoma |
| EGFR | MOL000098 | Quercetin | Coptidis Rhizoma |
| VEGFA | MOL000098 | Quercetin | Coptidis Rhizoma |
| BCL2 | MOL000098 | Quercetin | Coptidis Rhizoma |
| PLAU | MOL000098 | Quercetin | Coptidis Rhizoma |
| MMP2 | MOL000098 | Quercetin | Coptidis Rhizoma |
| MAPK1 | MOL000098 | Quercetin | Coptidis Rhizoma |
| EGF | MOL000098 | Quercetin | Coptidis Rhizoma |
| RB1 | MOL000098 | Quercetin | Coptidis Rhizoma |
| TNFAIP6 | MOL000098 | Quercetin | Coptidis Rhizoma |
| JUN | MOL000098 | Quercetin | Coptidis Rhizoma |
| IL6R | MOL000098 | Quercetin | Coptidis Rhizoma |
| TP53 | MOL000098 | Quercetin | Coptidis Rhizoma |
| POR | MOL000098 | Quercetin | Coptidis Rhizoma |
| ODC1 | MOL000098 | Quercetin | Coptidis Rhizoma |
| TOP1 | MOL000098 | Quercetin | Coptidis Rhizoma |
| SOD1 | MOL000098 | Quercetin | Coptidis Rhizoma |
| MMP1 | MOL000098 | Quercetin | Coptidis Rhizoma |
| CDK1 | MOL000098 | Quercetin | Coptidis Rhizoma |
| HSPA5 | MOL000098 | Quercetin | Coptidis Rhizoma |
| ACACA | MOL000098 | Quercetin | Coptidis Rhizoma |
| HMOX1 | MOL000098 | Quercetin | Coptidis Rhizoma |
| CYP3A4 | MOL000098 | Quercetin | Coptidis Rhizoma |
| CYP1A2 | MOL000098 | Quercetin | Coptidis Rhizoma |
| F3 | MOL000098 | Quercetin | Coptidis Rhizoma |
| GJA1 | MOL000098 | Quercetin | Coptidis Rhizoma |
| IL1B | MOL000098 | Quercetin | Coptidis Rhizoma |
| CCL2 | MOL000098 | Quercetin | Coptidis Rhizoma |
| SELE | MOL000098 | Quercetin | Coptidis Rhizoma |
| VCAM1 | MOL000098 | Quercetin | Coptidis Rhizoma |
| PTGER3 | MOL000098 | Quercetin | Coptidis Rhizoma |
| SULT1E1 | MOL000098 | Quercetin | Coptidis Rhizoma |
| IL2RA | MOL000098 | Quercetin | Coptidis Rhizoma |
| PLAT | MOL000098 | Quercetin | Coptidis Rhizoma |
| THBD | MOL000098 | Quercetin | Coptidis Rhizoma |
| COL1A1 | MOL000098 | Quercetin | Coptidis Rhizoma |
| IFNG | MOL000098 | Quercetin | Coptidis Rhizoma |
| ALOX5 | MOL000098 | Quercetin | Coptidis Rhizoma |
| MPO | MOL000098 | Quercetin | Coptidis Rhizoma |
| GSTP1 | MOL000098 | Quercetin | Coptidis Rhizoma |
| NQO1 | MOL000098 | Quercetin | Coptidis Rhizoma |
| AHR | MOL000098 | Quercetin | Coptidis Rhizoma |
| COL3A1 | MOL000098 | Quercetin | Coptidis Rhizoma |
| INSRR | MOL000098 | Quercetin | Coptidis Rhizoma |
| ACP3 | MOL000098 | Quercetin | Coptidis Rhizoma |
| CTSD | MOL000098 | Quercetin | Coptidis Rhizoma |
| PON1 | MOL000098 | Quercetin | Coptidis Rhizoma |
| GSTM1 | MOL000098 | Quercetin | Coptidis Rhizoma |
| GSTM2 | MOL000098 | Quercetin | Coptidis Rhizoma |
| GABRA1 | MOL000622 | Magnograndiolide | Coptidis Rhizoma |
| GRIA2 | MOL000622 | Magnograndiolide | Coptidis Rhizoma |
| NOS2 | MOL000785 | Palmatine | Coptidis Rhizoma |
| PTGS1 | MOL000785 | Palmatine | Coptidis Rhizoma |
| KCNH2 | MOL000785 | Palmatine | Coptidis Rhizoma |
| ESR1 | MOL000785 | Palmatine | Coptidis Rhizoma |
| AR | MOL000785 | Palmatine | Coptidis Rhizoma |
| SCN5A | MOL000785 | Palmatine | Coptidis Rhizoma |
| PTGS2 | MOL000785 | Palmatine | Coptidis Rhizoma |
| RXRA | MOL000785 | Palmatine | Coptidis Rhizoma |
| ADRB2 | MOL000785 | Palmatine | Coptidis Rhizoma |
| ESR2 | MOL000785 | Palmatine | Coptidis Rhizoma |
| HSP90AB1 | MOL000785 | Palmatine | Coptidis Rhizoma |
| PRSS1 | MOL000785 | Palmatine | Coptidis Rhizoma |
| NCOA2 | MOL000785 | Palmatine | Coptidis Rhizoma |
| CAMKMT | MOL000785 | Palmatine | Coptidis Rhizoma |
| CDK2 | MOL000785 | Palmatine | Coptidis Rhizoma |
| F7 | MOL000785 | Palmatine | Coptidis Rhizoma |
| NOS2 | MOL001454 | Berberine | Coptidis Rhizoma |
| PTGS1 | MOL001454 | Berberine | Coptidis Rhizoma |
| KCNH2 | MOL001454 | Berberine | Coptidis Rhizoma |
| ESR1 | MOL001454 | Berberine | Coptidis Rhizoma |
| AR | MOL001454 | Berberine | Coptidis Rhizoma |
| SCN5A | MOL001454 | Berberine | Coptidis Rhizoma |
| PTGS2 | MOL001454 | Berberine | Coptidis Rhizoma |
| RXRA | MOL001454 | Berberine | Coptidis Rhizoma |
| ADRB2 | MOL001454 | Berberine | Coptidis Rhizoma |
| HSP90AB1 | MOL001454 | Berberine | Coptidis Rhizoma |
| PRSS1 | MOL001454 | Berberine | Coptidis Rhizoma |
| NCOA2 | MOL001454 | Berberine | Coptidis Rhizoma |
| PDE10A | MOL001454 | Berberine | Coptidis Rhizoma |
| CAMKMT | MOL001454 | Berberine | Coptidis Rhizoma |
| NOS2 | MOL001458 | Coptisine | Coptidis Rhizoma |
| PTGS1 | MOL001458 | Coptisine | Coptidis Rhizoma |
| KCNH2 | MOL001458 | Coptisine | Coptidis Rhizoma |
| ESR1 | MOL001458 | Coptisine | Coptidis Rhizoma |
| AR | MOL001458 | Coptisine | Coptidis Rhizoma |
| SCN5A | MOL001458 | Coptisine | Coptidis Rhizoma |
| PTGS2 | MOL001458 | Coptisine | Coptidis Rhizoma |
| PRSS1 | MOL001458 | Coptisine | Coptidis Rhizoma |
| NOS2 | MOL002668 | Worenine | Coptidis Rhizoma |
| PTGS1 | MOL002668 | Worenine | Coptidis Rhizoma |
| ESR1 | MOL002668 | Worenine | Coptidis Rhizoma |
| AR | MOL002668 | Worenine | Coptidis Rhizoma |
| PTGS2 | MOL002668 | Worenine | Coptidis Rhizoma |
| CHEK1 | MOL002668 | Worenine | Coptidis Rhizoma |
| NOS2 | MOL002894 | Berberrubine | Coptidis Rhizoma |
| PTGS1 | MOL002894 | Berberrubine | Coptidis Rhizoma |
| KCNH2 | MOL002894 | Berberrubine | Coptidis Rhizoma |
| ESR1 | MOL002894 | Berberrubine | Coptidis Rhizoma |
| AR | MOL002894 | Berberrubine | Coptidis Rhizoma |
| SCN5A | MOL002894 | Berberrubine | Coptidis Rhizoma |
| PTGS2 | MOL002894 | Berberrubine | Coptidis Rhizoma |
| RXRA | MOL002894 | Berberrubine | Coptidis Rhizoma |
| PRSS1 | MOL002894 | Berberrubine | Coptidis Rhizoma |
| NCOA2 | MOL002894 | Berberrubine | Coptidis Rhizoma |
| CAMKMT | MOL002894 | Berberrubine | Coptidis Rhizoma |
| NOS2 | MOL002897 | Epiberberine | Coptidis Rhizoma |
| KCNH2 | MOL002897 | Epiberberine | Coptidis Rhizoma |
| ESR1 | MOL002897 | Epiberberine | Coptidis Rhizoma |
| AR | MOL002897 | Epiberberine | Coptidis Rhizoma |
| PTGS2 | MOL002897 | Epiberberine | Coptidis Rhizoma |
| RXRA | MOL002897 | Epiberberine | Coptidis Rhizoma |
| PRSS1 | MOL002897 | Epiberberine | Coptidis Rhizoma |
| NCOA2 | MOL002897 | Epiberberine | Coptidis Rhizoma |
| PDE10A | MOL002897 | Epiberberine | Coptidis Rhizoma |
| PTGS1 | MOL002903 | (R)-Canadine | Coptidis Rhizoma |
| CHRM3 | MOL002903 | (R)-Canadine | Coptidis Rhizoma |
| KCNH2 | MOL002903 | (R)-Canadine | Coptidis Rhizoma |
| CHRM1 | MOL002903 | (R)-Canadine | Coptidis Rhizoma |
| SCN5A | MOL002903 | (R)-Canadine | Coptidis Rhizoma |
| CHRM5 | MOL002903 | (R)-Canadine | Coptidis Rhizoma |
| PTGS2 | MOL002903 | (R)-Canadine | Coptidis Rhizoma |
| HTR3A | MOL002903 | (R)-Canadine | Coptidis Rhizoma |
| ADRA2C | MOL002903 | (R)-Canadine | Coptidis Rhizoma |
| CHRM4 | MOL002903 | (R)-Canadine | Coptidis Rhizoma |
| OPRD1 | MOL002903 | (R)-Canadine | Coptidis Rhizoma |
| ADRA1B | MOL002903 | (R)-Canadine | Coptidis Rhizoma |
| SLC6A3 | MOL002903 | (R)-Canadine | Coptidis Rhizoma |
| ADRB2 | MOL002903 | (R)-Canadine | Coptidis Rhizoma |
| ADRA1D | MOL002903 | (R)-Canadine | Coptidis Rhizoma |
| SLC6A4 | MOL002903 | (R)-Canadine | Coptidis Rhizoma |
| OPRM1 | MOL002903 | (R)-Canadine | Coptidis Rhizoma |
| HSP90AB1 | MOL002903 | (R)-Canadine | Coptidis Rhizoma |
| PDE10A | MOL002903 | (R)-Canadine | Coptidis Rhizoma |
| CAMKMT | MOL002903 | (R)-Canadine | Coptidis Rhizoma |
| DRD1 | MOL002903 | (R)-Canadine | Coptidis Rhizoma |
| DRD5 | MOL002903 | (R)-Canadine | Coptidis Rhizoma |
| RXRA | MOL002903 | (R)-Canadine | Coptidis Rhizoma |
| SLC6A2 | MOL002903 | (R)-Canadine | Coptidis Rhizoma |
| ADRA1A | MOL002903 | (R)-Canadine | Coptidis Rhizoma |
| CHRM2 | MOL002903 | (R)-Canadine | Coptidis Rhizoma |
| NOS2 | MOL002904 | Berlambine | Coptidis Rhizoma |
| PTGS1 | MOL002904 | Berlambine | Coptidis Rhizoma |
| CHRM3 | MOL002904 | Berlambine | Coptidis Rhizoma |
| KCNH2 | MOL002904 | Berlambine | Coptidis Rhizoma |
| AR | MOL002904 | Berlambine | Coptidis Rhizoma |
| SCN5A | MOL002904 | Berlambine | Coptidis Rhizoma |
| PTGS2 | MOL002904 | Berlambine | Coptidis Rhizoma |
| F7 | MOL002904 | Berlambine | Coptidis Rhizoma |
| RXRA | MOL002904 | Berlambine | Coptidis Rhizoma |
| ADRA1B | MOL002904 | Berlambine | Coptidis Rhizoma |
| ADRB2 | MOL002904 | Berlambine | Coptidis Rhizoma |
| ADRA1D | MOL002904 | Berlambine | Coptidis Rhizoma |
| HSP90AB1 | MOL002904 | Berlambine | Coptidis Rhizoma |
| PRSS1 | MOL002904 | Berlambine | Coptidis Rhizoma |
| NCOA2 | MOL002904 | Berlambine | Coptidis Rhizoma |
| CAMKMT | MOL002904 | Berlambine | Coptidis Rhizoma |
| NR3C2 | MOL002907 | Corchoroside A_qt | Coptidis Rhizoma |
| NCOA2 | MOL002907 | Corchoroside A_qt | Coptidis Rhizoma |

Table S3 Information of Disease–drug Action Targets

| Target Name | Mol ID | Mol Name | Drug |
| --- | --- | --- | --- |
| PTGS2 | MOL000358 | Beta-sitosterol | Aurantii Fructus |
|  | MOL002341 | Hesperetin | Aurantii Fructus |
|  | MOL005828 | Nobiletin | Aurantii Fructus |
|  | MOL013381 | Marmin | Aurantii Fructus |
|  | MOL000098 | Quercetin | Coptidis Rhizoma |
|  | MOL000785 | Palmatine | Coptidis Rhizoma |
|  | MOL001454 | Berberine | Coptidis Rhizoma |
|  | MOL001458 | Coptisine | Coptidis Rhizoma |
|  | MOL002668 | Worenine | Coptidis Rhizoma |
|  | MOL002894 | Berberrubine | Coptidis Rhizoma |
|  | MOL002897 | Epiberberine | Coptidis Rhizoma |
|  | MOL002903 | (R)-Canadine | Coptidis Rhizoma |
|  | MOL002904 | Berlambine | Coptidis Rhizoma |
| NOS2 | MOL005828 | Nobiletin | Aurantii Fructus |
|  | MOL000785 | Palmatine | Coptidis Rhizoma |
|  | MOL001454 | Berberine | Coptidis Rhizoma |
|  | MOL001458 | Coptisine | Coptidis Rhizoma |
|  | MOL002668 | Worenine | Coptidis Rhizoma |
|  | MOL002894 | Berberrubine | Coptidis Rhizoma |
|  | MOL002897 | Epiberberine | Coptidis Rhizoma |
|  | MOL002904 | Berlambine | Coptidis Rhizoma |
| OPRM1 | MOL000358 | Beta-sitosterol | Aurantii Fructus |
|  | MOL002903 | (R)-Canadine | Coptidis Rhizoma |
| PPARG | MOL005828 | Nobiletin | Aurantii Fructus |
|  | MOL000098 | Quercetin | Coptidis Rhizoma |
| SOD1 | MOL004328 | Naringenin | Aurantii Fructus |
|  | MOL000098 | Quercetin | Coptidis Rhizoma |
| JUN | MOL005828 | Nobiletin | Aurantii Fructus |
|  | MOL000098 | Quercetin | Coptidis Rhizoma |
| GSTP1 | MOL004328 | Naringenin | Aurantii Fructus |
|  | MOL000098 | Quercetin | Coptidis Rhizoma |
| MMP3 | MOL000098 | Quercetin | Coptidis Rhizoma |
| RB1 | MOL000098 | Quercetin | Coptidis Rhizoma |
| IL6R | MOL000098 | Quercetin | Coptidis Rhizoma |
| HMOX1 | MOL000098 | Quercetin | Coptidis Rhizoma |
| F3 | MOL000098 | Quercetin | Coptidis Rhizoma |
| IL1B | MOL000098 | Quercetin | Coptidis Rhizoma |
| CCL2 | MOL000098 | Quercetin | Coptidis Rhizoma |
| SELE | MOL000098 | Quercetin | Coptidis Rhizoma |
| VCAM1 | MOL000098 | Quercetin | Coptidis Rhizoma |
| IL2RA | MOL000098 | Quercetin | Coptidis Rhizoma |
| IFNG | MOL000098 | Quercetin | Coptidis Rhizoma |
| ALOX5 | MOL000098 | Quercetin | Coptidis Rhizoma |
| ABCC1 | MOL004328 | Naringenin | Aurantii Fructus |

Table S4 Information of some GO function analysis

| ONTOLOGY | ID | Description | geneID | pvalue |
| --- | --- | --- | --- | --- |
| BP | GO:0050727 | regulation of inflammatory response | PTGS2/PPARG/MMP3/RB1/SOD1/IL1B/SELE/IL2RA/IFNG/ALOX5/GSTP1/ABCC1 | 1.12E-15 |
| BP | GO:0002526 | acute inflammatory response | PTGS2/OPRM1/IL6R/F3/IL1B/VCAM1/GSTP1 | 1.75E-11 |
| BP | GO:0009410 | response to xenobiotic stimulus | NOS2/PTGS2/RB1/JUN/SOD1/HMOX1/IL1B/GSTP1/ABCC1 | 2.10E-10 |
| BP | GO:0032496 | response to lipopolysaccharide | NOS2/PTGS2/OPRM1/IL1B/CCL2/SELE/VCAM1/GSTP1 | 1.03E-09 |
| BP | GO:0002237 | response to molecule of bacterial origin | NOS2/PTGS2/OPRM1/IL1B/CCL2/SELE/VCAM1/GSTP1 | 1.65E-09 |
| BP | GO:0006979 | response to oxidative stress | PTGS2/MMP3/JUN/SOD1/HMOX1/ALOX5/GSTP1/ABCC1 | 7.16E-09 |
| BP | GO:1903039 | positive regulation of leukocyte cell-cell adhesion | IL1B/CCL2/SELE/VCAM1/IL2RA/IFNG/ALOX5 | 8.43E-09 |
| BP | GO:0150076 | neuroinflammatory response | PTGS2/MMP3/JUN/IL1B/IFNG | 1.11E-08 |
| BP | GO:1903131 | mononuclear cell differentiation | PPARG/JUN/IL6R/SOD1/IL1B/VCAM1/IL2RA/IFNG | 1.40E-08 |
| BP | GO:0001819 | positive regulation of cytokine production | NOS2/PTGS2/IL6R/SOD1/HMOX1/F3/IL1B/IFNG | 1.73E-08 |
| BP | GO:0048660 | regulation of smooth muscle cell proliferation | PTGS2/PPARG/JUN/IL6R/HMOX1/IFNG | 2.01E-08 |
| BP | GO:0019221 | cytokine-mediated signaling pathway | PPARG/IL6R/F3/IL1B/CCL2/IL2RA/IFNG/GSTP1 | 2.03E-08 |
| BP | GO:0048659 | smooth muscle cell proliferation | PTGS2/PPARG/JUN/IL6R/HMOX1/IFNG | 2.30E-08 |
| BP | GO:0022409 | positive regulation of cell-cell adhesion | IL1B/CCL2/SELE/VCAM1/IL2RA/IFNG/ALOX5 | 2.50E-08 |
| BP | GO:0001936 | regulation of endothelial cell proliferation | PPARG/JUN/HMOX1/F3/CCL2/ALOX5 | 2.63E-08 |
| BP | GO:0050728 | negative regulation of inflammatory response | PPARG/RB1/SOD1/IL2RA/ALOX5/GSTP1 | 2.71E-08 |
| BP | GO:0062197 | cellular response to chemical stress | PTGS2/MMP3/JUN/SOD1/HMOX1/ALOX5/ABCC1 | 4.01E-08 |
| BP | GO:0001935 | endothelial cell proliferation | PPARG/JUN/HMOX1/F3/CCL2/ALOX5 | 4.33E-08 |
| BP | GO:0032642 | regulation of chemokine production | IL6R/HMOX1/IL1B/IFNG/GSTP1 | 4.92E-08 |
| BP | GO:0032602 | chemokine production | IL6R/HMOX1/IL1B/IFNG/GSTP1 | 5.18E-08 |
| BP | GO:1903037 | regulation of leukocyte cell-cell adhesion | IL1B/CCL2/SELE/VCAM1/IL2RA/IFNG/ALOX5 | 7.36E-08 |
| BP | GO:0050900 | leukocyte migration | IL6R/HMOX1/IL1B/CCL2/SELE/VCAM1/ALOX5 | 1.06E-07 |
| BP | GO:0007159 | leukocyte cell-cell adhesion | IL1B/CCL2/SELE/VCAM1/IL2RA/IFNG/ALOX5 | 1.39E-07 |
| BP | GO:0050678 | regulation of epithelial cell proliferation | PPARG/RB1/JUN/HMOX1/F3/CCL2/ALOX5 | 1.39E-07 |
| BP | GO:0051101 | regulation of DNA binding | PPARG/RB1/JUN/HMOX1/IFNG | 1.42E-07 |
| BP | GO:0033002 | muscle cell proliferation | PTGS2/PPARG/JUN/IL6R/HMOX1/IFNG | 1.56E-07 |
| BP | GO:0006690 | icosanoid metabolic process | PTGS2/IL1B/ALOX5/GSTP1/ABCC1 | 1.74E-07 |
| BP | GO:0050708 | regulation of protein secretion | NOS2/OPRM1/PPARG/IL1B/IFNG/ALOX5 | 1.97E-07 |
| BP | GO:0032102 | negative regulation of response to external stimulus | PPARG/RB1/SOD1/CCL2/IL2RA/ALOX5/GSTP1 | 1.98E-07 |
| BP | GO:0031348 | negative regulation of defense response | PPARG/RB1/SOD1/IL2RA/ALOX5/GSTP1 | 2.75E-07 |
| BP | GO:0032103 | positive regulation of response to external stimulus | PTGS2/OPRM1/IL6R/F3/IL1B/IFNG/ABCC1 | 3.02E-07 |
| BP | GO:0071715 | icosanoid transport | NOS2/PTGS2/IL1B/ABCC1 | 3.23E-07 |
| BP | GO:0023061 | signal release | NOS2/PTGS2/OPRM1/PPARG/IL1B/IFNG/ALOX5 | 3.29E-07 |
| BP | GO:0034599 | cellular response to oxidative stress | MMP3/JUN/SOD1/HMOX1/ALOX5/ABCC1 | 3.85E-07 |
| BP | GO:0050673 | epithelial cell proliferation | PPARG/RB1/JUN/HMOX1/F3/CCL2/ALOX5 | 3.85E-07 |
| BP | GO:0045785 | positive regulation of cell adhesion | IL1B/CCL2/SELE/VCAM1/IL2RA/IFNG/ALOX5 | 4.01E-07 |
| BP | GO:0022407 | regulation of cell-cell adhesion | IL1B/CCL2/SELE/VCAM1/IL2RA/IFNG/ALOX5 | 4.36E-07 |
| BP | GO:0062013 | positive regulation of small molecule metabolic process | NOS2/PTGS2/PPARG/IL1B/IFNG | 4.43E-07 |
| BP | GO:0090594 | inflammatory response to wounding | PPARG/HMOX1/ALOX5 | 6.82E-07 |
| BP | GO:0060326 | cell chemotaxis | IL6R/IL1B/CCL2/VCAM1/ALOX5/ABCC1 | 7.00E-07 |
| BP | GO:0032722 | positive regulation of chemokine production | IL6R/HMOX1/IL1B/IFNG | 7.99E-07 |
| BP | GO:0032310 | prostaglandin secretion | NOS2/PTGS2/IL1B | 8.18E-07 |
| BP | GO:0001933 | negative regulation of protein phosphorylation | PPARG/RB1/JUN/IL1B/IFNG/GSTP1 | 9.81E-07 |
| BP | GO:0062012 | regulation of small molecule metabolic process | NOS2/PTGS2/PPARG/SOD1/IL1B/IFNG | 9.98E-07 |
| BP | GO:0050766 | positive regulation of phagocytosis | SOD1/IL1B/CCL2/IFNG | 1.00E-06 |
| BP | GO:0071466 | cellular response to xenobiotic stimulus | NOS2/RB1/IL1B/GSTP1/ABCC1 | 1.11E-06 |
| BP | GO:0006809 | nitric oxide biosynthetic process | NOS2/PTGS2/IL1B/IFNG | 1.11E-06 |
| BP | GO:0015732 | prostaglandin transport | NOS2/PTGS2/IL1B | 1.14E-06 |
| BP | GO:0008217 | regulation of blood pressure | NOS2/PTGS2/PPARG/SOD1/HMOX1 | 1.37E-06 |
| BP | GO:0009306 | protein secretion | NOS2/OPRM1/PPARG/IL1B/IFNG/ALOX5 | 1.39E-06 |
| BP | GO:0035592 | establishment of protein localization to extracellular region | NOS2/OPRM1/PPARG/IL1B/IFNG/ALOX5 | 1.42E-06 |
| BP | GO:0046209 | nitric oxide metabolic process | NOS2/PTGS2/IL1B/IFNG | 1.51E-06 |
| BP | GO:0051767 | nitric-oxide synthase biosynthetic process | CCL2/IFNG/GSTP1 | 1.54E-06 |
| BP | GO:0051769 | regulation of nitric-oxide synthase biosynthetic process | CCL2/IFNG/GSTP1 | 1.54E-06 |
| BP | GO:2000310 | regulation of NMDA receptor activity | OPRM1/CCL2/IFNG | 1.54E-06 |
| BP | GO:2001057 | reactive nitrogen species metabolic process | NOS2/PTGS2/IL1B/IFNG | 1.59E-06 |
| BP | GO:0070997 | neuron death | RB1/JUN/SOD1/HMOX1/CCL2/IFNG | 1.61E-06 |
| BP | GO:0071692 | protein localization to extracellular region | NOS2/OPRM1/PPARG/IL1B/IFNG/ALOX5 | 1.61E-06 |
| BP | GO:2001233 | regulation of apoptotic signaling pathway | PTGS2/RB1/SOD1/HMOX1/IL1B/GSTP1 | 1.77E-06 |
| BP | GO:0050863 | regulation of T cell activation | SOD1/IL1B/CCL2/VCAM1/IL2RA/IFNG | 1.82E-06 |
| BP | GO:0042326 | negative regulation of phosphorylation | PPARG/RB1/JUN/IL1B/IFNG/GSTP1 | 1.97E-06 |
| BP | GO:0006469 | negative regulation of protein kinase activity | PPARG/RB1/IL1B/IFNG/GSTP1 | 2.51E-06 |
| BP | GO:0097191 | extrinsic apoptotic signaling pathway | IL6R/HMOX1/IL1B/IFNG/GSTP1 | 3.00E-06 |
| BP | GO:0048661 | positive regulation of smooth muscle cell proliferation | PTGS2/JUN/IL6R/HMOX1 | 3.09E-06 |
| BP | GO:0032755 | positive regulation of interleukin-6 production | NOS2/IL6R/IL1B/IFNG | 3.22E-06 |
| BP | GO:0071241 | cellular response to inorganic substance | PTGS2/MMP3/JUN/SOD1/HMOX1 | 3.34E-06 |
| BP | GO:0030098 | lymphocyte differentiation | IL6R/SOD1/IL1B/VCAM1/IL2RA/IFNG | 3.41E-06 |
| BP | GO:2001234 | negative regulation of apoptotic signaling pathway | PTGS2/RB1/HMOX1/IL1B/GSTP1 | 3.63E-06 |
| BP | GO:0046883 | regulation of hormone secretion | NOS2/PPARG/IL1B/IFNG/ALOX5 | 3.71E-06 |
| BP | GO:0050764 | regulation of phagocytosis | SOD1/IL1B/CCL2/IFNG | 3.77E-06 |
| BP | GO:0072593 | reactive oxygen species metabolic process | NOS2/MMP3/SOD1/ALOX5/GSTP1 | 3.79E-06 |
| BP | GO:0033673 | negative regulation of kinase activity | PPARG/RB1/IL1B/IFNG/GSTP1 | 4.20E-06 |
| BP | GO:0045936 | negative regulation of phosphate metabolic process | PPARG/RB1/JUN/IL1B/IFNG/GSTP1 | 4.52E-06 |
| BP | GO:0010563 | negative regulation of phosphorus metabolic process | PPARG/RB1/JUN/IL1B/IFNG/GSTP1 | 4.58E-06 |
| BP | GO:0033198 | response to ATP | PTGS2/SOD1/IL1B | 4.93E-06 |
| BP | GO:0050870 | positive regulation of T cell activation | IL1B/CCL2/VCAM1/IL2RA/IFNG | 5.22E-06 |
| BP | GO:0015833 | peptide transport | NOS2/IL1B/IFNG/ALOX5/ABCC1 | 5.54E-06 |
| BP | GO:0034612 | response to tumor necrosis factor | PTGS2/CCL2/SELE/VCAM1/GSTP1 | 5.54E-06 |
| BP | GO:0051402 | neuron apoptotic process | RB1/JUN/SOD1/HMOX1/CCL2 | 5.64E-06 |
| BP | GO:0033559 | unsaturated fatty acid metabolic process | PTGS2/IL1B/ALOX5/GSTP1 | 5.84E-06 |
| BP | GO:0071346 | cellular response to interferon-gamma | NOS2/PPARG/CCL2/IFNG | 6.47E-06 |
| BP | GO:0031667 | response to nutrient levels | PTGS2/OPRM1/PPARG/SOD1/HMOX1/VCAM1 | 6.92E-06 |
| BP | GO:0071901 | negative regulation of protein serine/threonine kinase activity | PPARG/RB1/IL1B/GSTP1 | 7.39E-06 |
| BP | GO:0071276 | cellular response to cadmium ion | JUN/SOD1/HMOX1 | 7.69E-06 |
| BP | GO:0051348 | negative regulation of transferase activity | PPARG/RB1/IL1B/IFNG/GSTP1 | 8.29E-06 |
| BP | GO:0045923 | positive regulation of fatty acid metabolic process | PTGS2/PPARG/IL1B | 9.76E-06 |
| BP | GO:0150077 | regulation of neuroinflammatory response | PTGS2/MMP3/IL1B | 1.05E-05 |
| BP | GO:0046879 | hormone secretion | NOS2/PPARG/IL1B/IFNG/ALOX5 | 1.07E-05 |
| BP | GO:0010632 | regulation of epithelial cell migration | PTGS2/PPARG/JUN/HMOX1/IFNG | 1.09E-05 |
| BP | GO:0045429 | positive regulation of nitric oxide biosynthetic process | PTGS2/IL1B/IFNG | 1.13E-05 |
| BP | GO:0001666 | response to hypoxia | NOS2/PTGS2/PPARG/HMOX1/VCAM1 | 1.16E-05 |
| BP | GO:0030217 | T cell differentiation | IL6R/SOD1/IL1B/IL2RA/IFNG | 1.16E-05 |
| BP | GO:0042886 | amide transport | NOS2/IL1B/IFNG/ALOX5/ABCC1 | 1.16E-05 |
| BP | GO:0009914 | hormone transport | NOS2/PPARG/IL1B/IFNG/ALOX5 | 1.24E-05 |
| BP | GO:0034341 | response to interferon-gamma | NOS2/PPARG/CCL2/IFNG | 1.27E-05 |
| BP | GO:1904407 | positive regulation of nitric oxide metabolic process | PTGS2/IL1B/IFNG | 1.30E-05 |
| BP | GO:0036293 | response to decreased oxygen levels | NOS2/PTGS2/PPARG/HMOX1/VCAM1 | 1.43E-05 |
| BP | GO:0042063 | gliogenesis | RB1/SOD1/IL1B/CCL2/IFNG | 1.45E-05 |
| BP | GO:0070372 | regulation of ERK1 and ERK2 cascade | OPRM1/JUN/IL1B/CCL2/GSTP1 | 1.57E-05 |
| BP | GO:0140353 | lipid export from cell | NOS2/PTGS2/IL1B | 1.59E-05 |
| CC | GO:0005901 | caveola | PTGS2/HMOX1/SELE | 7.34E-05 |
| CC | GO:0009897 | external side of plasma membrane | IL6R/F3/SELE/VCAM1/IL2RA | 7.73E-05 |
| CC | GO:0044853 | plasma membrane raft | PTGS2/HMOX1/SELE | 0.000190282 |
| CC | GO:0045121 | membrane raft | PTGS2/OPRM1/HMOX1/SELE | 0.000280042 |
| CC | GO:0098857 | membrane microdomain | PTGS2/OPRM1/HMOX1/SELE | 0.000283327 |
| CC | GO:0032839 | dendrite cytoplasm | OPRM1/SOD1 | 0.000439846 |
| CC | GO:0090575 | RNA polymerase II transcription regulator complex | PPARG/RB1/JUN | 0.001893346 |
| CC | GO:0120111 | neuron projection cytoplasm | OPRM1/SOD1 | 0.003655976 |
| CC | GO:0031970 | organelle envelope lumen | SOD1/ALOX5 | 0.003980481 |
| CC | GO:0030863 | cortical cytoskeleton | NOS2/SELE | 0.005217624 |
| CC | GO:1904813 | ficolin-1-rich granule lumen | ALOX5/GSTP1 | 0.006820437 |
| CC | GO:0042383 | sarcolemma | OPRM1/VCAM1 | 0.008151264 |
| CC | GO:0005777 | peroxisome | NOS2/SOD1 | 0.008857145 |
| CC | GO:0042579 | microbody | NOS2/SOD1 | 0.008857145 |
| CC | GO:0045177 | apical part of cell | IL6R/VCAM1/ABCC1 | 0.009006769 |
| CC | GO:0005641 | nuclear envelope lumen | ALOX5 | 0.010022719 |
| CC | GO:0005677 | chromatin silencing complex | RB1 | 0.013010876 |
| CC | GO:1905286 | serine-type peptidase complex | F3 | 0.013010876 |
| CC | GO:0101002 | ficolin-1-rich granule | ALOX5/GSTP1 | 0.014674531 |
| MF | GO:0031406 | carboxylic acid binding | NOS2/PPARG/SELE/GSTP1 | 3.09E-05 |
| MF | GO:0016209 | antioxidant activity | PTGS2/SOD1/GSTP1 | 0.000101951 |
| MF | GO:0004896 | cytokine receptor activity | IL6R/F3/IL2RA | 0.00015093 |
| MF | GO:0005126 | cytokine receptor binding | IL6R/IL1B/CCL2/IFNG | 0.000189176 |
| MF | GO:0016702 | oxidoreductase activity, acting on single donors with incorporation of molecular oxygen, incorporation of two atoms of oxygen | PTGS2/ALOX5 | 0.000304334 |
| MF | GO:0070412 | R-SMAD binding | PPARG/JUN | 0.000304334 |
| MF | GO:0016701 | oxidoreductase activity, acting on single donors with incorporation of molecular oxygen | PTGS2/ALOX5 | 0.000330583 |
| MF | GO:0020037 | heme binding | NOS2/PTGS2/HMOX1 | 0.000435516 |
| MF | GO:0140375 | immune receptor activity | IL6R/F3/IL2RA | 0.00052314 |
| MF | GO:0046906 | tetrapyrrole binding | NOS2/PTGS2/HMOX1 | 0.000533521 |
| MF | GO:0016705 | oxidoreductase activity, acting on paired donors, with incorporation or reduction of molecular oxygen | NOS2/PTGS2/HMOX1 | 0.000909119 |
| MF | GO:0005504 | fatty acid binding | PPARG/GSTP1 | 0.001275814 |
| MF | GO:0004601 | peroxidase activity | PTGS2/GSTP1 | 0.00160477 |
| MF | GO:0016684 | oxidoreductase activity, acting on peroxide as acceptor | PTGS2/GSTP1 | 0.001722482 |
| MF | GO:0005125 | cytokine activity | IL1B/CCL2/IFNG | 0.002035563 |
| MF | GO:0046332 | SMAD binding | PPARG/JUN | 0.003197077 |
| MF | GO:0033293 | monocarboxylic acid binding | PPARG/GSTP1 | 0.003442681 |
| MF | GO:0051213 | dioxygenase activity | PTGS2/ALOX5 | 0.004510349 |
| MF | GO:0004497 | monooxygenase activity | NOS2/HMOX1 | 0.005606747 |
| MF | GO:0061629 | RNA polymerase II-specific DNA-binding transcription factor binding | PPARG/RB1/JUN | 0.006088517 |
| MF | GO:0019838 | growth factor binding | IL6R/IL2RA | 0.008887996 |
| MF | GO:0043177 | organic acid binding | NOS2/PPARG | 0.009545785 |
| MF | GO:0070851 | growth factor receptor binding | IL6R/IL1B | 0.009950647 |
| MF | GO:0019955 | cytokine binding | IL6R/IL2RA | 0.010502247 |
| MF | GO:0004955 | prostaglandin receptor activity | PPARG | 0.01080049 |
| MF | GO:0043225 | ATPase-coupled inorganic anion transmembrane transporter activity | ABCC1 | 0.01080049 |
| MF | GO:0004954 | prostanoid receptor activity | PPARG | 0.011874423 |
| MF | GO:0035673 | oligopeptide transmembrane transporter activity | ABCC1 | 0.011874423 |
| MF | GO:0005178 | integrin binding | IL1B/VCAM1 | 0.012236772 |
| MF | GO:0097677 | STAT family protein binding | PPARG | 0.012947248 |
| MF | GO:0140297 | DNA-binding transcription factor binding | PPARG/RB1/JUN | 0.014071257 |
| MF | GO:0004252 | serine-type endopeptidase activity | MMP3/F3 | 0.015057386 |
| MF | GO:0008559 | ABC-type xenobiotic transporter activity | ABCC1 | 0.015089579 |
| MF | GO:0015562 | efflux transmembrane transporter activity | ABCC1 | 0.015089579 |
| MF | GO:0048018 | receptor ligand activity | IL1B/CCL2/IFNG | 0.015287802 |
| MF | GO:0030546 | signaling receptor activator activity | IL1B/CCL2/IFNG | 0.015875536 |
| MF | GO:0004953 | icosanoid receptor activity | PPARG | 0.016159088 |
| MF | GO:0010181 | FMN binding | NOS2 | 0.016159088 |
| MF | GO:0036041 | long-chain fatty acid binding | PPARG | 0.016159088 |
| MF | GO:0046965 | nuclear retinoid X receptor binding | PPARG | 0.016159088 |
| MF | GO:0070492 | oligosaccharide binding | SELE | 0.016159088 |
| MF | GO:1904680 | peptide transmembrane transporter activity | ABCC1 | 0.016159088 |
| MF | GO:0008022 | protein C-terminus binding | OPRM1/PPARG | 0.016223333 |
| MF | GO:0016641 | oxidoreductase activity, acting on the CH-NH2 group of donors, oxygen as acceptor | VCAM1 | 0.017227493 |
| MF | GO:0008236 | serine-type peptidase activity | MMP3/F3 | 0.01813162 |
| MF | GO:0005149 | interleukin-1 receptor binding | IL1B | 0.018294796 |
| MF | GO:0035497 | cAMP response element binding | JUN | 0.018294796 |
| MF | GO:0017171 | serine hydrolase activity | MMP3/F3 | 0.018848229 |
| MF | GO:0016638 | oxidoreductase activity, acting on the CH-NH2 group of donors | VCAM1 | 0.021490102 |
| MF | GO:0031681 | G-protein beta-subunit binding | OPRM1 | 0.022553006 |
| MF | GO:0004602 | glutathione peroxidase activity | GSTP1 | 0.023614814 |
| MF | GO:0033691 | sialic acid binding | SELE | 0.023614814 |
| MF | GO:0043274 | phospholipase binding | SELE | 0.024675525 |
| MF | GO:0042974 | nuclear retinoic acid receptor binding | PPARG | 0.025735142 |
| MF | GO:0001965 | G-protein alpha-subunit binding | OPRM1 | 0.027851096 |
| MF | GO:0004364 | glutathione transferase activity | GSTP1 | 0.027851096 |
| MF | GO:0072349 | modified amino acid transmembrane transporter activity | ABCC1 | 0.027851096 |
| MF | GO:0051393 | alpha-actinin binding | PPARG | 0.029962684 |
| MF | GO:0042910 | xenobiotic transmembrane transporter activity | ABCC1 | 0.033121898 |
| MF | GO:0090482 | vitamin transmembrane transporter activity | ABCC1 | 0.034172795 |
| MF | GO:0042923 | neuropeptide binding | OPRM1 | 0.035222607 |
| MF | GO:0097718 | disordered domain specific binding | RB1 | 0.037318981 |
| MF | GO:0042805 | actinin binding | PPARG | 0.038365544 |
| MF | GO:0042887 | amide transmembrane transporter activity | ABCC1 | 0.038365544 |
| MF | GO:0031625 | ubiquitin protein ligase binding | RB1/JUN | 0.041119101 |
| MF | GO:0016709 | oxidoreductase activity, acting on paired donors, with incorporation or reduction of molecular oxygen, NAD(P)H as one donor, and incorporation of one atom of oxygen | NOS2 | 0.042540997 |
| MF | GO:0016712 | oxidoreductase activity, acting on paired donors, with incorporation or reduction of molecular oxygen, reduced flavin or flavoprotein as one donor, and incorporation of one atom of oxygen | HMOX1 | 0.042540997 |
| MF | GO:0044389 | ubiquitin-like protein ligase binding | RB1/JUN | 0.045955867 |
| MF | GO:0005245 | voltage-gated calcium channel activity | OPRM1 | 0.046699223 |
| MF | GO:0001227 | DNA-binding transcription repressor activity, RNA polymerase II-specific | PPARG/JUN | 0.047262712 |
| MF | GO:0001217 | DNA-binding transcription repressor activity | PPARG/JUN | 0.048318159 |
| MF | GO:0008188 | neuropeptide receptor activity | OPRM1 | 0.048771897 |
| MF | GO:0042277 | peptide binding | OPRM1/PPARG | 0.049115502 |
| MF | GO:0048020 | CCR chemokine receptor binding | CCL2 | 0.049806628 |

Only the top 100 pieces of BP information are displayed

Table S5 Information of KEGG function analysis

| ID | Description | geneID | P-value |
| --- | --- | --- | --- |
| hsa05418 | Fluid shear stress and atherosclerosis | JUN/HMOX1/IL1B/CCL2/SELE/VCAM1/IFNG/GSTP1 | 4.61E-10 |
| hsa04668 | TNF signaling pathway | PTGS2/MMP3/JUN/IL1B/CCL2/SELE/VCAM1 | 4.48E-09 |
| hsa04657 | IL-17 signaling pathway | PTGS2/MMP3/JUN/IL1B/CCL2/IFNG | 5.46E-08 |
| hsa04933 | AGE-RAGE signaling pathway in diabetic complications | JUN/F3/IL1B/CCL2/SELE/VCAM1 | 7.93E-08 |
| hsa05144 | Malaria | IL1B/CCL2/SELE/VCAM1/IFNG | 8.50E-08 |
| hsa05417 | Lipid and atherosclerosis | PPARG/MMP3/JUN/IL1B/CCL2/SELE/VCAM1 | 3.62E-07 |
| hsa05140 | Leishmaniasis | NOS2/PTGS2/JUN/IL1B/IFNG | 7.61E-07 |
| hsa05143 | African trypanosomiasis | IL1B/SELE/VCAM1/IFNG | 1.42E-06 |
| hsa05323 | Rheumatoid arthritis | MMP3/JUN/IL1B/CCL2/IFNG | 1.96E-06 |
| hsa05142 | Chagas disease | NOS2/JUN/IL1B/CCL2/IFNG | 3.09E-06 |
| hsa04659 | Th17 cell differentiation | JUN/IL6R/IL1B/IL2RA/IFNG | 4.10E-06 |
| hsa04066 | HIF-1 signaling pathway | NOS2/IL6R/HMOX1/IFNG | 0.000107565 |
| hsa05163 | Human cytomegalovirus infection | PTGS2/RB1/IL6R/IL1B/CCL2 | 0.000142027 |
| hsa05171 | Coronavirus disease - COVID-19 | MMP3/JUN/IL6R/IL1B/CCL2 | 0.000164034 |
| hsa04380 | Osteoclast differentiation | PPARG/JUN/IL1B/IFNG | 0.000200393 |
| hsa04932 | Non-alcoholic fatty liver disease | PPARG/JUN/IL6R/IL1B | 0.000417004 |
| hsa05321 | Inflammatory bowel disease | JUN/IL1B/IFNG | 0.00044892 |
| hsa04060 | Cytokine-cytokine receptor interaction | IL6R/IL1B/CCL2/IL2RA/IFNG | 0.000516487 |
| hsa05133 | Pertussis | NOS2/JUN/IL1B | 0.000710626 |
| hsa04658 | Th1 and Th2 cell differentiation | JUN/IL2RA/IFNG | 0.00123918 |
| hsa05222 | Small cell lung cancer | NOS2/PTGS2/RB1 | 0.00123918 |
| hsa05215 | Prostate cancer | MMP3/RB1/GSTP1 | 0.001443967 |
| hsa04640 | Hematopoietic cell lineage | IL6R/IL1B/IL2RA | 0.001531493 |
| hsa04061 | Viral protein interaction with cytokine and cytokine receptor | IL6R/CCL2/IL2RA | 0.001576482 |
| hsa05146 | Amoebiasis | NOS2/IL1B/IFNG | 0.001668939 |
| hsa04064 | NF-kappa B signaling pathway | PTGS2/IL1B/VCAM1 | 0.001764741 |
| hsa04625 | C-type lectin receptor signaling pathway | PTGS2/JUN/IL1B | 0.001764741 |
| hsa05145 | Toxoplasmosis | NOS2/IFNG/ALOX5 | 0.002182276 |
| hsa01523 | Antifolate resistance | IL1B/ABCC1 | 0.002217875 |
| hsa05135 | Yersinia infection | JUN/IL1B/CCL2 | 0.003865587 |
| hsa05162 | Measles | JUN/IL1B/IL2RA | 0.004026509 |
| hsa05332 | Graft-versus-host disease | IL1B/IFNG | 0.004315963 |
| hsa04940 | Type I diabetes mellitus | IL1B/IFNG | 0.004520102 |
| hsa04913 | Ovarian steroidogenesis | PTGS2/ALOX5 | 0.006310449 |
| hsa04630 | JAK-STAT signaling pathway | IL6R/IL2RA/IFNG | 0.006608388 |
| hsa05225 | Hepatocellular carcinoma | RB1/HMOX1/GSTP1 | 0.006831028 |
| hsa05164 | Influenza A | IL1B/CCL2/IFNG | 0.007173364 |
| hsa05152 | Tuberculosis | NOS2/IL1B/IFNG | 0.008261408 |
| hsa00590 | Arachidonic acid metabolism | PTGS2/ALOX5 | 0.008930347 |
| hsa04621 | NOD-like receptor signaling pathway | JUN/IL1B/CCL2 | 0.009038364 |
| hsa05167 | Kaposi sarcoma-associated herpesvirus infection | PTGS2/RB1/JUN | 0.010139581 |
| hsa05204 | Chemical carcinogenesis - DNA adducts | PTGS2/GSTP1 | 0.011319916 |
| hsa05166 | Human T-cell leukemia virus 1 infection | RB1/JUN/IL2RA | 0.014596119 |
| hsa05208 | Chemical carcinogenesis - reactive oxygen species | JUN/SOD1/HMOX1 | 0.014772927 |
| hsa04146 | Peroxisome | NOS2/SOD1 | 0.015733538 |
| hsa05235 | PD-L1 expression and PD-1 checkpoint pathway in cancer | JUN/IFNG | 0.018370438 |
| hsa01522 | Endocrine resistance | RB1/JUN | 0.022016347 |
| hsa05022 | Pathways of neurodegeneration - multiple diseases | NOS2/PTGS2/SOD1/IL1B | 0.023318033 |
| hsa04620 | Toll-like receptor signaling pathway | JUN/IL1B | 0.024601099 |
| hsa04660 | T cell receptor signaling pathway | JUN/IFNG | 0.024601099 |
